# Effect of a physical activity and behaviour maintenance programme on functional mobility decline in older adults: the REACT (Retirement in Action) randomised controlled trial

**DOI:** 10.1016/S2468-2667(22)00004-4

**Published:** 2022-03-21

**Authors:** Afroditi Stathi, Colin J Greaves, Janice L Thompson, Janet Withall, Peter Ladlow, Gordon Taylor, Antonieta Medina-Lara, Tristan Snowsill, Selena Gray, Colin Green, Heidi Johansen-Berg, Claire E Sexton, James L J Bilzon, Jolanthe deKoning, Jessica C Bollen, Sarah J Moorlock, Max J Western, Naiara Demnitz, Poppy Seager, Jack M Guralnik, W Jack Rejeski, Melvyn Hillsdon, Kenneth R Fox

**Affiliations:** a School of Sport, Exercise and Rehabilitation Sciences, University of Birmingham, Birmingham, UK; b Academic Department of Military Rehabilitation (ADMR), Defence Medical Rehabilitation Centre (DMRC), Stanford Hall, Loughborough, UK; c College of Medicine and Health, University of Exeter, Exeter, UK; d College of Life and Environmental Sciences, University of Exeter, Exeter, UK; e Faculty of Health and Applied Sciences, University of the West of England (UWE) Bristol, Bristol, UK; f Oxford Centre for Functional MRI of the Brain, Wellcome Centre for Integrative Neuroimaging, John Radcliffe Hospital, University of Oxford, Oxford, UK; g Oxford Centre for Human Brain Activity, Wellcome Centre for Integrative Neuroimaging, Department of Psychiatry, University of Oxford, Warneford Hospital, Oxford, UK; h Global Brain Health Institute, Memory and Aging Center, Department of Neurology, University of California San Francisco, San Francisco, CA, USA; i Department for Health, University of Bath, Bath, UK; j Birmingham Clinical Trials Unit, University of Birmingham, Birmingham, UK; k Danish Research Centre for Magnetic Resonance, Centre for Functional Diagnostic Imaging and Research, Copenhagen University Hospital –Amager and Hvidovre, Hvidovre, Denmark; l Department of Epidemiology and Public Health, University of Maryland School of Medicine, Baltimore, MD, USA; m Department of Health and Exercise Science, Wake Forest University, Winston-Salem, NC, USA; n Centre for Exercise, Nutrition and Health Sciences, School for Policy Studies, University of Bristol, Bristol, UK

## Abstract

**Background:**

Mobility limitations in old age can greatly reduce quality of life, generate substantial health and social care costs, and increase mortality. Through the Retirement in Action (REACT) trial, we aimed to establish whether a community-based active ageing intervention could prevent decline in lower limb physical functioning in older adults already at increased risk of mobility limitation.

**Methods:**

In this pragmatic, multicentre, two-arm, single-blind, parallel-group, randomised, controlled trial, we recruited older adults (aged 65 years or older and who are not in full-time employment) with reduced lower limb physical functioning (Short Physical Performance Battery [SPPB] score 4–9) from 35 primary care practices across three sites (Bristol and Bath; Birmingham; and Devon) in England. Participants were randomly assigned to receive brief advice (three healthy ageing education sessions) or a 12-month, group-based, multimodal physical activity (64 1-h exercise sessions) and behavioural maintenance (21 45-min sessions) programme delivered by charity and community or leisure centre staff in local communities. Randomisation was stratified by site and adopted a minimisation approach to balance groups by age, sex, and SPPB score, using a centralised, online, randomisation algorithm. Researchers involved in data collection and analysis were masked but participants were not because of the nature of the intervention. The primary outcome was change in SPPB score at 24 months, analysed by intention to treat. This trial is registered with ISRCTN, ISRCTN45627165.

**Findings:**

Between June 20, 2016, and Oct 30, 2017, 777 participants (mean age 77·6 [SD 6·8] years; 66% female; mean SPPB score 7·37 [1·56]) were randomly assigned to the intervention (n=410) and control (n=367) groups. Primary outcome data at 24 months were provided by 628 (81%) participants (294 in the control group and 334 in the intervention group). At the 24-month follow-up, the SPPB score (adjusted for baseline SPPB score, age, sex, study site, and exercise group) was significantly greater in the intervention group (mean 8·08 [SD 2·87]) than in the control group (mean 7·59 [2·61]), with an adjusted mean difference of 0·49 (95% CI 0·06–0·92; p=0·014), which is just below our predefined clinically meaningful difference of 0·50. One adverse event was related to the intervention; the most common unrelated adverse events were heart conditions, strokes, and falls.

**Interpretation:**

For older adults at risk of mobility limitations, the REACT intervention showed that a 12-month physical activity and behavioural maintenance programme could help prevent decline in physical function over a 24-month period.

**Funding:**

National Institute for Health Research Public Health Research Programme (13/164/51).

## Graphical abstract

**Figure:**
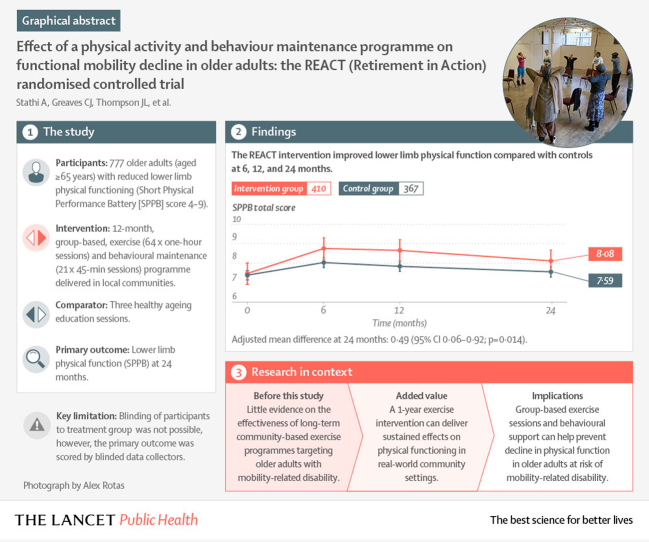

## Introduction

With increasing age, there is a population-wide decline in physical function.^1^, ^2^ 44% of state pension-age adults in the UK are classified as disabled.^3^ The most common form of disability is mobility-related disability (67%), a major public health issue that is considerably reducing the independence and quality of life of older adults while also contributing to high health and social care costs and increased mortality.^4^, ^5^ Reduced gait speed and low levels of physical activity are key markers of frailty, which itself increases the pressure on health-care systems worldwide. Pressure on health-care systems is further exacerbated by the rapid expansion of the older population.^6^

In older people, there is strong evidence of a positive effect from regular physical activity on lower limb physical functioning,^7^, ^8^ the ability to live independently in the community, reduced hospital admissions, and mortality.^9^, ^10^ Despite these substantial benefits, people become less physically active and more sedentary as they age, with only 12% of UK adults aged 65 years and older meeting UK physical activity guidelines.^11^ Sedentary behaviours are even more prevalent in socioeconomically deprived parts of the population,^12^ which is a key driver of health inequalities.^13^

**Research in context**

**Evidence before this study**

We searched Google Scholar, the Cochrane Database Of Systematic Reviews, the National Institute for Health Research (NIHR) library, and PubMed for systematic reviews and meta-analyses of trials of exercise-based interventions to improve physical function in older people published in English from inception to Jan 31, 2014, using the terms “older people”, “physical activity”, “physical function”, and “randomised controlled trials”. We found strong evidence for a positive effect of physical activity on physical functioning, independent living, mobility-related disability, falls, hospital admissions, and mortality. Despite these substantial benefits, the evidence indicates that people become less physically active and more sedentary as they age. Low levels of physical activity are even more prevalent in socioeconomically deprived sectors of the population. The US Lifestyle Interventions and Independence for Elders efficacy trial showed that physical activity can reduce the risk of developing major mobility-related disability. However, we found very little evidence regarding the effectiveness and cost-effectiveness of long-term exercise programmes (ie, those lasting at least 1 year) administered at a community level, in a real-world setting, and that are tailored for a UK population of older adults at risk of mobility-related disability. Furthermore, we found no long-term, community-based interventions that reported effects for 12 months or more after the intervention.

**Added value of this study**

This study adds robust evidence that a 1-year exercise intervention can improve physical functioning in real-world community settings in the UK, with benefits that are sustained for at least 24 months.

**Implications of all the available evidence**

Group-based physical activity sessions, with strong social and behavioural change elements, are an effective approach to maintaining good physical function in older adults who are at risk of increasing mobility-related disability.

The Lifestyle Interventions and Independence for Elders (LIFE) study was a landmark US-based efficacy trial that showed that a physical activity intervention designed to improve lower limb strength, balance, and stamina can reduce the risk of developing major mobility-related disability by 18% and persistent mobility-related disability by 28%.^14^ Major mobility-related disability was defined as the inability to complete a 400-metre walk test within 15 min without sitting and without the help of another person or walker (objectively assessed)—a well validated and important clinical and public health outcome in older people, associated with mortality, cardiovascular disease, mobility limitation, and disability.^15^ However, the LIFE intervention was resource-intensive and there was no long-term follow-up after the intervention. Since the publication of that clinical trial, the challenge has been to develop affordable and scalable physical activity interventions that target mobility and that are suitable for delivery in a range of community contexts; the effects of these interventions should also be maintained in the long term.

The Retirement in Action (REACT) study is a pragmatic effectiveness trial designed to assess the short-term and long-term effect of a real-world community-based exercise programme and social and behaviour maintenance support for older adults at increased risk of mobility limitations in the UK. We hypothesised that participants allocated to the 12-month REACT intervention group would have significantly better lower limb physical function at 24-month follow-up than participants allocated to the control group.

## Methods

### Study design

REACT was a pragmatic, multicentre, two-arm, single-blind, parallel-group, randomised, controlled trial, with an internal pilot phase, incorporating comprehensive process and economic evaluations. Pragmatic trials are designed to assess the effectiveness of an intervention as it would be delivered in the real world, rather than under highly controlled conditions.^16^ Ethical approval was provided by the National Health Service (NHS) South East Coast–Surrey Research Ethics Committee (15/LO/2082). The full protocol and intervention description are published elsewhere.^17^

### Participants

Full inclusion and exclusion criteria for participants are detailed in the study protocol.^17^ Briefly, community-dwelling adults aged 65 years or older who are not in full-time employment and who scored between 4 and 9 (inclusive) on the Short Physical Performance Battery (SPPB) were recruited primarily from 35 primary care practices in urban and semi-rural locations across three sites in England: Bath and Bristol; Birmingham; and Devon. The SPPB criteria identified people who have mobility limitations but are still ambulatory, and included people classified as physically frail (SPPB 4–7) and pre-frail (SPPB 8–9) by the European Medicines Agency.^18^ We excluded people who were unable to walk across a room without the help of another person, living in residential care, awaiting hip or knee surgery, or receiving radiation therapy or chemotherapy, along with people who had had recent heart or spinal surgery or had an illness that would prevent participation, such as those with severe arthritis, diagnosed moderate-to-severe dementia, severe kidney disease, unstable heart disease, and severe psychiatric illness.

Recruitment was primarily done through invitation letters from general practitioners (GPs),^19^ and advertised by third sector or charity organisations, local media (articles and low-cost advertising in local newspapers, magazines, radio, and at community events), and word of mouth. This process enabled the recruitment of a socioeconomically and ethnically diverse sample that included participants from urban, rural, and semi-rural locations. Written informed consent was obtained from all study participants. The full recruitment strategy and recruitment results are published elsewhere.^19^

### Randomisation and masking

Participants meeting the study inclusion criteria were randomly assigned to either the physical activity and behavioural maintenance intervention group or the control group using a secure, centralised, randomisation website hosted by the Peninsula Clinical Trial Unit. Randomisation was done using a minimisation algorithm to balance groups by study site, age group (65–74 years *vs* ≥75 years), sex, and baseline functional ability (SPPB 4–7 *vs* 8–9).

Participant data were entered into the randomisation website, which generated a unique study identification number and randomisation code for each participant. The researchers at each site who contacted participants to inform them of their allocation were not involved in any primary outcome testing at follow-up. During the internal pilot phase, the randomisation ratio was 2:1 (favouring the intervention) to enable feasibility testing of intervention engagement and set-up or delivery processes as early as possible. No changes were made to the intervention during or after the pilot phase. The main trial randomisation ratio was 1:1. 39 couples or pairs of close friends who were both eligible were randomised together to minimise contamination between study groups.

Researchers collecting and entering the primary outcome data (SPPB scores), the senior research team, and the trial statistician were masked to group allocation to minimise bias.^20^ Participants and intervention provider staff were not masked to the intervention allocation because of the nature of the intervention. The participant invitation to the assessment included a request not to reveal group allocation to the researchers and this request was reiterated on arrival. The chief investigator was unmasked when needed to allow assessment of serious adverse events.

### Procedures

Participants in the intervention group received a manualised 12-month exercise and behavioural maintenance programme, designed for delivery in leisure or community centres by qualified exercise professionals. A comprehensive manual outlining the content and structure of types of exercise to be delivered, methods for progression, safety considerations, methods for tailoring exercises and progression to individual capabilities, and the behavioural maintenance sessions was distributed to the session leaders before their training. REACT session leaders were qualified to at least Register of Exercise Professionals level 3 (Exercise Referral Diploma or equivalent) and were experienced in delivering safe and effective exercise sessions to older adults. The exercise sessions were designed to improve lower limb muscle strength and balance. The 1-h exercise sessions were delivered twice a week for 12 weeks, reduced to once a week for a further 40 weeks (64 sessions in total over 12 months) to groups of around 15 participants. Despite being delivered in a group setting, exercise programmes were personalised on the basis of participants' functional status and goals, using the Rate of Perceived Exertion scale (a 15-point numerical scale ranging from 6 to 20).^21^ During the 12-month exercise intervention, strength-based exercises were prescribed to reflect intensities rated from moderate to vigorous (ie, 11–16). Towards the end of each session, games-based activities lasting 15–20 min were delivered at intensities from light to moderate (ie, 8–13). By accommodating for daily fluctuations in residual muscle soreness or fatigue, rate of perceived exertion methods encourage more tolerable adjustments to individual training loads on a session-by-session basis^22^—an important consideration for the long-term adherence to any exercise intervention for older adults.^23^ This individualised approach to exercise prescription enabled each participant to progress at their own pace.

The exercise sessions were each followed by 20 min of refreshments and socialising to promote session attendance and contribute to participants' social wellbeing. After 9 weeks, the behavioural maintenance programme commenced as a 45-min session delivered once a week (immediately following the exercise class). The maintenance sessions were designed to provide physical activity and health information and emphasised long-term maintenance of an active lifestyle, including the promotion of ongoing engagement in exercise classes, home-based exercise, neighbourhood walking, and active travel. They incorporated behaviour change techniques derived from social cognitive theory, self-determination theory,^24^, ^25^ and the Skills for Maintenance (SkiM) model.^26^ These techniques included building intrinsic motivation; making realistic plans for sustainable activity; pre-empting and overcoming barriers; maximising enjoyment, social interaction, and group identity; and engaging external social support and using self-monitoring and self-regulatory techniques to support the maintenance of behaviour change. From week 25 of the intervention, the behaviour maintenance programme was reduced to one meeting per month for the remainder of the programme (six further meetings in total, resulting in 21 meetings overall). Further details of the intervention are provided in the published protocol.^17^

Participants in the control group were invited to attend three workshops lasting 60–90 min each, delivered before the 6-month, 12-month, and 24-month assessments. The workshops covered healthy ageing topics with no physical activity content (eg, healthy eating, dealing with dementia, and volunteering). Transport for attending either the intervention sessions or the control group workshops was not provided.

### Outcomes

Outcomes were measured at baseline and at 6, 12, and 24 months after randomisation in a group setting at local community centres, as per the assessment schedule (appendix pp 1–2). Home assessments were offered if participants could not attend at community centres.

The primary outcome was the SPPB score at 24 months.^27^ The SPPB measures normal walking speed over 4 metres, time to complete five repeated rises from a chair, and completion of three standing balance tasks of increasing difficulty. Each measure was scored from 0 (inability to complete the test) to 4 (best performance) and the sum of the three component scores was calculated (0–12). Therefore, the SPPB assesses the performance of basic physical functions relevant to everyday life. Several studies using both anchor-based and distribution-based approaches have shown the correlation of changes in SPPB score with changes in the ability to walk one block or a quarter of a mile, or to climb a flight of stairs (anchor-based) in the general population of older adults and in specific subgroups based on race, BMI, self-reported health, and common chronic conditions.^28^, ^29^, ^30^, ^31^ SPPB provides a meaningful way to represent important aspects of lower limb physical functioning in older people and is used widely as a primary outcome in clinical trials focusing on the effect of exercise on physical function.

Secondary outcomes are fully described in the protocol.^17^ In brief, these were: (1) change in minutes of mean daily moderate-to-vigorous-intensity physical activity (MVPA), assessed by a wrist-worn accelerometer (GENEActiv Original; Activinsights, Kimbolton, UK) and calculated as the sum of all activity above the 100 milligravitational unit (mg) threshold (total minutes of MVPA) in total and that which was accumulated in bouts of at least 10 min (bouted MVPA); (2) mean daily sedentary time, calculated as all the time spent below the 40-mg threshold minus accelerometer-estimated sleep time,^32^ and mean number of breaks in sedentary time, calculated as the frequency of active bouts that lasted 60 s or longer per day; (3) self-reported physical activity, assessed by the Physical Activity Scale for the Elderly (PASE) questionnaire; (4) the short form of the Mobility Assessment Tool; (5) self-reported adherence to government guidelines on muscle-strengthening activity, assessed by the Muscle-Strengthening Exercise Questionnaire (appendix pp 24–41); (6) dominant hand-grip strength, assessed by dynamometer; (7) a falls inventory, including number of falls and fall-related injuries; (8) cognitive function, using the UK Biobank Healthy Minds Questionnaire to assess simple processing speed, episodic memory, fluid intelligence, working memory, visual attention, and complex processing speed; and a brief questionnaire, including validated measures of (9) social wellbeing (Ageing Well Profile); (10) sleep quality (Sleep Condition Indicator); (11) hip, knee, and ankle joint pain (Western Ontario and McMaster Universities Osteoarthritis Index); (12) health-related quality of life (36-item short-form survey [SF-36] and Euroquol 5-dimension questionnaire [EQ-5D]); and (13) a one-item loneliness scale (appendix pp 24–41). A full cost-effectiveness analysis, including within-trial changes in EQ-5D and quality-adjusted life years, and a model-based lifetime analysis is reported separately.^33^ A mixed-methods process evaluation, including an assessment of intervention fidelity (ie, of the quality of delivery), will also be presented elsewhere.

At baseline, we collected demographic information on age, sex, ethnicity, BMI, cognitive impairment (using the Montreal Cognitive Assessment),^34^ education level, area deprivation (using the Multiple Deprivation Index, which was derived from postcodes), caring responsibilities, marital status, home ownership, and number of chronic illnesses. At all follow-up time points, we asked participants to report any other physical activity classes they had commenced (classes or groups).

Session attendance data were collected for the intervention group by the exercise instructor. In the statistical analysis plan (before analysis), we defined good attendance as attending at least 75% of intervention sessions and a minimum required dose as being at least 50% of intervention sessions. The quality of intervention delivery was assessed by: (1) direct observation of at least one structured exercise session at each provider site; and (2) a checklist rating of a purposive sample of audio recordings of the health behaviour maintenance sessions.^17^

Reports of adverse events were sought at each follow-up assessment point by research staff. In the intervention group, they were also reported by exercise session leaders. All serious adverse events were reported regardless of relatedness; non-serious adverse events (regardless of relatedness) were not reported.

### Statistical analysis

The power calculation for the primary outcome (SPPB score) at 24 months was based on detecting a plausible and clinically meaningful change in SPPB scores of at least 0·5 points,^14^, ^35^ an expected SD for change in SPPB scores from baseline to 2 years of 2·2,^12^ a two-sided significance level of 0·05, and an expected cumulative loss to follow-up of 12·5% per year. To provide 90% power, we required a sample size of 384 participants per group (768 in total) at baseline and a drop-out rate at 24 months of 25% or less.

Analyses were prespecified in the published protocol.^17^ The primary outcome analysis was done while masked to group allocation and included all participants in the groups to which they were randomly assigned and all available data. The model was adjusted for the four stratification variables (baseline SPPB score, age, sex, and study site). In addition, we adjusted the estimates for clustering by exercise group within the intervention group, using the approach recommended by Flight and colleagues.^36^ Therefore, the analysis used a mixed linear model. For participants in the intervention group, a random intercept term was included to account for the group they belonged to. Participants in the control group were entered as individual groups, each of size one. Age group and sex were included as fixed effects, study site as a random effect, baseline functional ability as a covariate, and the study group (intervention or control) as a fixed factor. All data were analysed at the level of the individual participant. The mixed linear model was implemented in Stata SE (version 15.0) using the mixed command. All available data were used in the analysis.

Secondary outcome analyses were done using the same approach as for the primary analysis (excluding the sensitivity analyses), with separate analyses at each timepoint (6, 12, and 24 months), adjusted for the baseline values of the outcome, using linear or logistic regression models for continuous or binary outcomes as appropriate.

In an exploratory sensitivity analysis, several predefined factors were entered as covariates in the regression model to examine their interaction with the primary outcome. These were: comorbidity levels at baseline (none or one *vs* two or more chronic medical conditions); socioeconomic subgroups (using education, home ownership, and quintiles of area deprivation); age categories (65–74 years *vs* ≥75 years); sex; study site (Bath and Bristol *vs* Birmingham *vs* Devon); history of falls (recorded fall or not during the 6 months before baseline); and the uptake of any co-interventions during the 24-month study period. To examine the association between dose (group session attendance) and response (SPPB outcome at 24 months), we did subgroup analyses comparing participants attending at least 50% and those attending at least 75% of the group sessions with (all) controls. Further sensitivity analyses used multiple imputation to estimate the effect of missing data (intermittent missingness and all loss to follow-up) on the primary analysis (estimates based on baseline age, sex, study group, SPPB score, BMI, study site, education quintile, and deprivation quintile) and repeated the primary analysis without accounting for clustering by exercise group within the intervention group. All secondary and sensitivity analyses were prespecified and were done using Stata (version 17.0). Due to an administrative error, two participants allocated to the control group were given the intervention and one participant allocated to the intervention group was given the control treatment. The analysis treated these participants according to their original allocation (on the basis of intention to treat).

A Trial Steering Committee, with advice from a Data Monitoring and Ethics Committee, oversaw the study. This trial is registered with ISRCTN, number ISRCTN45627165.

### Role of the funding source

The funder approved the study design but had no role in data collection, data analysis, data interpretation, or the writing of the report. AS and GT had full access to all the data in the study; AS had final responsibility for the decision to submit for publication.

## Results

**Figure 1:**
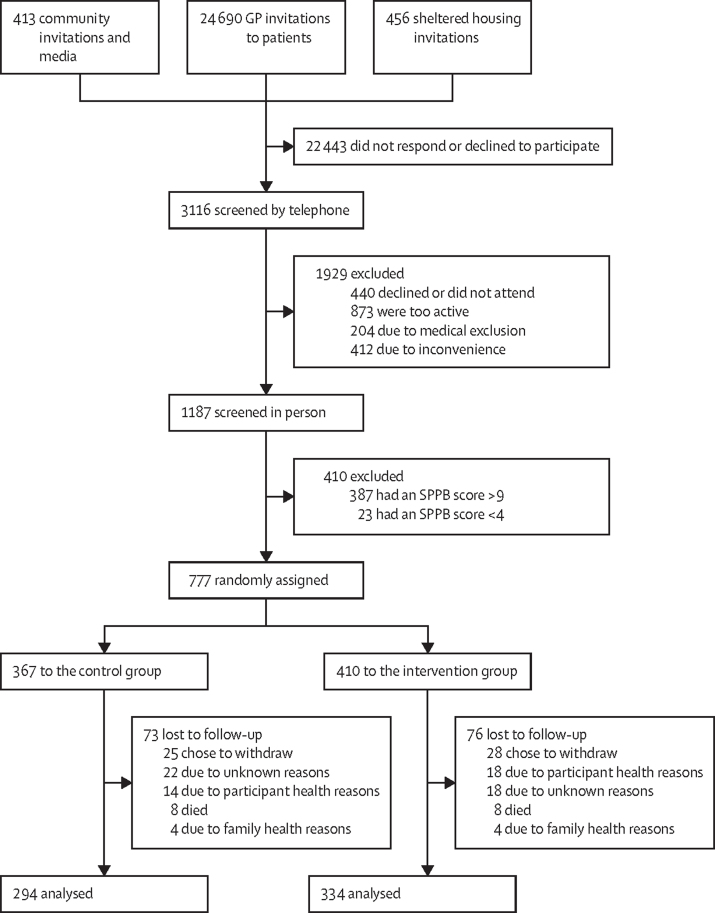
Trial profile GP=general practitioner. SPPB=Short Physical Performance Battery.

Between March 11, 2016, and Oct 19, 2017, 3116 people were screened by telephone (of whom 1077 were not eligible and 852 declined to participate further); 1187 individuals attended for baseline screening. Of these, 804 were found to be eligible and 777 were randomly assigned to either the control group (n=367) or the intervention group (n=410) between June 20, 2016, and Oct 30, 2017. Of the randomly assigned participants, 628 (81%) were included in the primary analysis at 24 months (294 [80%] from the control group and 334 [81%] from the intervention group; figure 1).

Baseline characteristics were similar between the two study groups (table 1), although there was a higher prevalence of multimorbidity in the intervention group (47%) than in the control group (39%). We recruited 138 participants to the internal pilot study between June 20 and Sept 30, 2016. No changes to the intervention protocol were made during or following the pilot study and data collection was completed on Oct 28, 2019. Data on recruitment yields and sample demographics in relation to the UK population older than 65 years of age have been published elsewhere.^17^ These data showed that the sample was broadly representative of the UK population older than 65 years of age in terms of sex, ethnicity, and area deprivation, although with some under-representation of men (34% *vs* 46% in the UK population older than 65 years of age).

**Table 1:** Baseline characteristics of trial participants

		**Control group (n=367)**	**Intervention group (n=410)**
Age, years		77·3 (6·64)	77·8 (6·93)
Sex			
	Female	241 (66%)	273 (67%)
	Male	126 (34%)	137 (33%)
Ethnicity			
	Caucasian or White	352 (96%)	387 (94%)
	African or Caribbean	9 (2%)	14 (3%)
	Asian	4 (1%)	5 (1%)
	Other or mixed	2 (1%)	4 (1%)
BMI, kg/m^2^		29·34 (5·51; 363)	29·20 (5·67; 404)
Cognitive impairment, MoCA score		24·29 (3·62; 354)	24·45 (3·70; 399)
Highest education level			
	Less than secondary	33/366 (9%)	31 (8%)
	Completed secondary	154/366 (42%)	142 (35%)
	Some college or vocational training	89/366 (24%)	117 (29%)
	College or university degree	73/366 (20%)	89 (22%)
	Graduate degree or higher	18/366 (5%)	31 (8%)
Index of Multiple Deprivation			
	Quintile 1	43 (12%)	43 (10%)
	Quintile 2	73 (20%)	83 (20%)
	Quintile 3	70 (19%)	89 (22%)
	Quintile 4	74 (20%)	82 (20%)
	Quintile 5	107 (29%)	113 (28%)
Caring responsibilities			
	Yes	37/310 (12%)	49/340 (14%)
	No	273/310 (88%)	291/340 (86%)
Marital status			
	Married or living with partner	158/313 (50%)	176/340 (52%)
	Widowed	90/313 (29%)	110/340 (32%)
	Divorced or separated	48/313 (15%)	31/340 (9%)
	Single and never married	17/313 (5%)	22/340 (6%)
	Other	0	1/340 (<1%)
Home ownership			
	Own home	259/312 (83%)	294/340 (86%)
	Renting or other	53/312 (17%)	46/340 (14%)
Number of chronic illnesses			
	None	90/360 (25%)	83/404 (21%)
	One	129/360 (36%)	131/404 (32%)
	Two or more	141/360 (39%)	190/404 (47%)
SPPB total score		7·36 (1·54; 367)	7·38 (1·58; 410)
	MVPA, min per day*	5·80 (8·62; 330)	5·94 (8·91; 374)
	Unbouted MVPA, min per day†	58·82 (32·18; 330)	55·10 (29·86; 374)
	Very low physical activity and sedentary time, min per day‡	804 (91·66; 318)	804 (91·52; 362)
	Breaks in sedentary time, N per day	43·23 (13·40; 328)	43·53 (13·36; 375)
PASE questionnaire score		119·90 (57·61; 359)	112·33 (58·13; 400)
Mobility Assessment Tool-short form score		49·89 (8·88; 357)	49·06 (9·75; 403)
Muscle-Strengthening Exercise Questionnaire score		3·18 (2·12; 338)	2·90 (2·01; 388)
Hand-grip strength, kg		24·92 (8·66; 361)	24·68 (8·49; 404)
	Number of falls in past 6 months	0·72 (1·15; 359)	0·69 (1·08; 401)
	Fall-related injury in past 6 months	45/355 (13%)	56/399 (14%)
UK Biobank Healthy Minds Questionnaire			
	Simple processing speed, ms	866·92 (277·42; 337)	865·70 (282·35; 383)
	Fluid intelligence score	3·60 (1·70; 332)	3·75 (1·59; 377)
	Executive function score	59 849·78 (31733·12; 254)	61 269·89 (38594·83; 283)
	Working memory 1 score	4·29 (1·44; 335)	4·37 (1·46; 382)
	Working memory 2 score	14·09 (6·43; 336)	13·73 (6·14; 383)
	Episodic memory score	5·94 (4·80; 333)	6·08 (4·29; 377)
Social wellbeing subscale score of Ageing Well Profile		23·92 (7·30; 347)	23·91 (6·74; 387)
Sleep Condition Indicator score		21·95 (7·90; 333)	22·53 (7·55; 342)
WOMAC score for pain		10·12 (3·77; 351)	9·73 (3·94; 399)
36-item short-form survey			
	Physical component score	30·01 (10·61; 392)	29·70 (10·96; 353)
	Mental component score	53·77 (8·66; 392)	54·55 (8·33; 353)
EUROQUOL-5 dimensions score		0·68 (0·17; 352)	0·69 (0·16; 357)
One-item loneliness scale score		135/361 (37%)	135/403 (33%)

Data are presented as mean (SD), mean (SD; N), n (%), or n/N (%), unless otherwise stated. mg=milligravitational unit. MoCA=Montreal Cognitive Impairment. MVPA=moderate-to-vigorous-intensity physical activity. PASE=Physical Activity Scale for the Elderly. SPPB=Short Physical Performance Battery. WOMAC=Western Ontario and McMaster Universities Arthritis Index.

* Time spent at >100 mg in at least 10-min bouts.

† All time spent at >100 mg.

‡ Excluding sleep.

At the 24-month follow-up, the mean SPPB score (adjusted for baseline SPPB score, age, sex, study site, and exercise group) was significantly greater in the intervention group (mean score 8·08 [SD 2·87]) than in the control group (mean score 7·59 [2·61]), with an adjusted mean difference of 0·49 (95% CI 0·06–0·92; p=0·014; table 2), which was on the border of our predefined, clinically meaningful difference of 0·50. Of the 410 participants allocated to the intervention group, 66 (16%) did not attend any intervention sessions (non-starters), 78 (19%) attended less than 50% of the sessions offered, 82 (20%) attended 50–74% of sessions, and 138 (45%) attended 75% or more. For all participants in the intervention group (including the non-starters), the mean percentage of sessions attended was 56·8% (95% CI 53·6–60·1). For participants in the intervention group who engaged with the programme (starters only), the mean percentage of sessions attended was 67·7% (65·1–70·4). An association between dose (group session attendance) and response (SPPB outcome at 24 months) was observed (appendix p 5), with an adjusted mean SPPB score difference of 0·64 (95% CI 0·23–1·05; p=0·0028) for those attending at least 50% of intervention sessions and 0·81 (0·38–1·23; p=0·0008) for those attending at least 75% of sessions. Only one instance of unmasking was reported during the collection of data at 24 months.

**Table 2:** Primary and secondary outcomes at 24 months

		**Control group (n=294)**	**Intervention group (n=334)**	**Estimated mean difference (95% CI)**	p value*
**Primary outcome**					
SPPB total score		7·59 (2·61)	8·08 (2·87)	0·49 (0·06 to 0·92)	0·014
**Secondary outcomes**					
MVPA, min per day†		4·50 (6·61; 250)	5·15 (5·99; 290)	0·65 (−0·48 to 1·78)	0·26
Unbouted MVPA, min per day‡		48·76 (19·48; 250)	51·22 (17·20; 290)	2·46 (−0·52 to 5·44)	0·11
Very low physical activity and sedentary time, min per day§		798 (65·80; 249)	804 (64·04; 287)	6·43 (−4·81 to 17·67)	0·26
Breaks in sedentary time, N per day		42·33 (13·54; 248)	40·76 (13·21; 287)	−1·57 (−3·89 to 0·75)	0·18
PASE questionnaire score		113·17 (52·10; 301)	123·90 (49·79; 328)	10·73 (2·62 to 18·84)	0·010
Mobility Assessment Tool-short form score		47·96 (8·13; 289)	49·99 (8·96; 319)	2·03 (0·66 to 3·40)	0·0042
Muscle-Strengthening Exercise Questionnaire score		3·18 (1·88; 276)	3·86 (2·30; 307)	0·68 (0·33 to 1·02)	0·0006
Hand-grip strength, kg		23·43 (4·08; 291)	23·74 (3·86; 328)	0·31 (−0·33 to 0·94)	0·34
Falls inventory					
	Number of falls in past 6 months	0·73 (1·05; 300)	0·70 (1·05; 330)	−0·02 (−0·19 to 0·14)	0·77
	Fall-related injury in past 6 months	51 (17·2; 297)	57 (17·5; 326)	0·3 (−5·92 to 6·46)¶	0·81
UK Biobank Healthy Minds Questionnaire					
	Simple processing speed, ms	811·28 (240·15; 264)	801·67 (246·72; 286)	−9·61 (−52·47 to 33·24)	0·66
	Fluid intelligence score	4·03 (1·41; 262)	4·19 (1·61; 282)	0·16 (−0·11 to 0·43)	0·23
	Executive function score	64 770·62 (38677·48; 210)	58 515·77 (35648·79; 236)	−6254·85 (−13498·22 to 988·52)	0·090
	Working memory 1 score	4·59 (1·29; 263)	4·46 (1·22; 282)	−0·13 (−0·35 to 0·06)	0·26
	Working memory 2 score	14·27 (5·24; 264)	14·62 (5·15; 285)	0·36 (−0·56 to 1·28)	0·44
	Episodic memory score	5·84 (4·19; 263)	5·36 (6·85; 286)	−0·48 (−1·49 to 0·53)	0·35
Social wellbeing subscale score of Ageing Well Profile		24·68 (5·85; 295)	24·88 (7·07; 306)	0·20 (−0·84 to 1·24)	0·70
Sleep Condition Indicator score		21·97 (6·10; 285)	22·50 (6·65; 311)	0·53 (−0·49 to 1·54)	0·31
WOMAC score for pain		10·20 (3·28; 290)	9·63 (3·95; 324)	−0·57 (−1·15 to 0)	0·052
SF-36					
	Physical component	29·38 (9·39; 295)	30·84 (10·04; 306)	1·46 (−0·09 to 3·01)	0·065
	Mental component	54·73 (7·64; 295)	54·33 (9·18; 306)	−0·40 (−1·78 to 0·98)	0·56
EUROQUOL-5 dimensions score		0·67 (0·16; 302)	0·69 (0·16; 330)	0·02 (−0·01 to 0·04)	0·22
One-item loneliness scale score		107 (35·7; 300)	110 (33·3; 330)	0·037 (−0·064 to 0·074)¶	0·91

Mean (SD) or mean (SD; N), unless otherwise stated.

* Adjusted for site, exercise group (within the intervention group), age group, sex, and baseline SPPB score.

† Time spent at >100 mg in at least 10-min bouts.

‡ All time spent at >100 mg.

§ Excluding sleep.

¶ Adjusted estimate and 95% CI for the between group percentage difference.

Primary and secondary outcomes at 24 months are presented in table 2 and all outcomes at 6 and 12 months are reported in the appendix (pp 3–4). SPPB scores were significantly higher in the intervention group than in the control group at 6 months (adjusted mean difference 0·68 [95% CI 0·39–0·96]; p=0·0009) and 12 months (0·77 [0·40–1·14]; p=0·0010). Self-reported physical activity was significantly higher in the intervention group at 6 months (adjusted mean difference in PASE score of 16·3 [95% CI 6·78–25·9]; p=0·0010), 12 months (10·8 [3·18–18·5]; p=0·0060), and 24 months (10·7 [2·62–18·8]; p=0·010). Self-reported engagement in muscle-strengthening exercise showed a similar pattern, with highly significant differences at all three follow-up times (p=0·0004, p=0·0004, and p=0·0006). Accelerometer data indicated a substantial difference favouring the intervention group at 12 months for total MVPA (adjusted mean difference 3·11 mins per day [95% CI 0·00–6·23]; p=0·050) and MVPA accumulated in bouts of at least 10 mins (1·24 mins per day [0·22–2·26]; p=0·018). Significant differences favouring the intervention group were also observed in the SF-36 physical component score (at 6 and 12 months), hand grip strength (at 12 months), EQ-5D-5L (at 6 months), and the short form of the Mobility Assessment Tool (MAT-SF) self-reported scale for lower limb physical functioning (at 6, 12, and 24 months). No significant differences were observed between groups for the other secondary outcomes.

**Figure 2:**
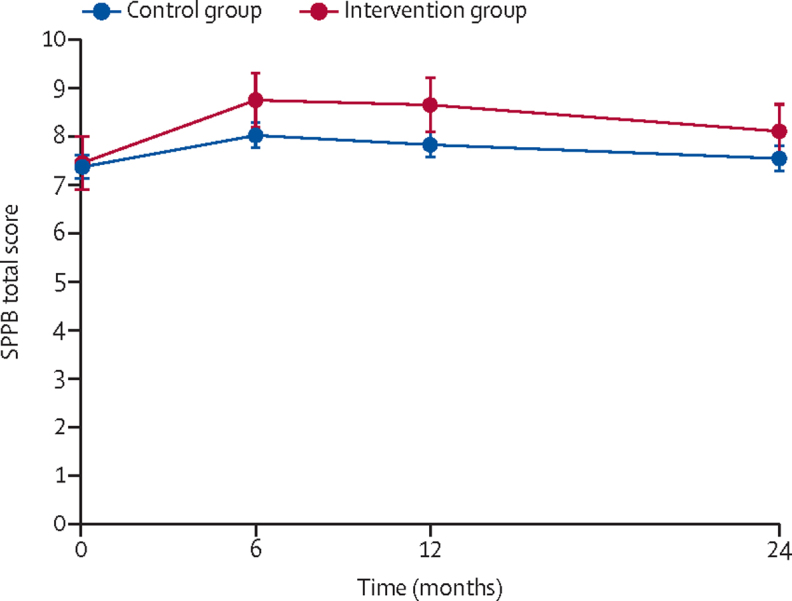
Predicted margins for SBBP total score (with 95% CIs) across all timepoints SPPB=Short Physical Performance Battery.

Sensitivity analyses that included the imputation of missing values and the fitting of a repeated measures model (figure 2; appendix p 9) and that did not adjust for clustering by exercise group did not substantially change the results (appendix p 5). Subgroup analyses according to education and deprivation level (key populations relevant for health inequality) and other characteristics found no significant interactions with study group, indicating no evidence of disparities between our pre-identified subgroups in the way the intervention worked (appendix pp 6–8).

During the study, 93 events were classified as serious adverse events (34 in the control group and 59 in the intervention group). The most common serious adverse events were heart conditions, stroke, and falls. Only one (a hip fracture from a fall caused by a chair breaking during an exercise session in the intervention group) was deemed to be related to the study (appendix pp 10–13).

In terms of intervention delivery, we found that, although the exercise sessions were delivered as intended, there was scope for improvement in the delivery of the health behaviour maintenance sessions, particularly in terms of the intended intervention processes for: monitoring progress; action planning; managing setbacks or problem solving; and supporting relatedness.

## Discussion

Older adults with mobility limitations who received the 12-month REACT intervention had significant improvements in lower limb physical function compared with control participants at 6, 12, and 24 months (12 months after the end of the intervention) of follow-up, indicating a sustained benefit over time (figure 2). These improvements were on the border of our predefined minimum clinically meaningful difference of 0·50. Higher intervention effects were associated with increased attendance to the programme group sessions.

The baseline SPPB scores were almost identical to the LIFE study population,^14^ enabling comparison; the observed difference in SPBB score of 0·49 at 24 months was 3 times larger than the between-group difference reported in the LIFE trial. In the LIFE trial, this smaller difference in SPPB scores was sufficient to reduce the subsequent risk of major mobility-related disability (defined as the objectively assessed inability to walk 400 meters) by 18% and the risk of persistent mobility-related disability (defined as two consecutive major mobility-related disability assessments or assessment of major mobility-related disability followed by death) by 28%. The ability to walk a distance of 400 metres strongly relates to maintenance of independent living.^15^ These effects from smaller changes in SPPB scores in the LIFE study suggest that the minimum clinically meaningful difference in SPPB scores might be lower than the difference of 0·50 used to calculate the sample size in this study. Indeed, other evidence suggests that changes in SPPB score of 0·28 or more are meaningful in frail or pre-frail older adults (SPPB score 4–9).^29^

At the completion of the intervention (12 months after baseline), significant differences were observed in the SF-36 physical component score, MAT-SF score, MVPA, self-reported physical activity, adherence to muscle-strengthening exercises, and hand-grip strength. These results are consistent with the idea that the intervention increased engagement in muscle strengthening, balance, and endurance exercises that mediated the observed effects on physical functioning.

The increase in MVPA (9 min per week bouted, or 22 min per week unbouted) is small, although it is worth noting that any increase in MVPA can have effects on health,^37^ and these increases must be set against the very low levels of initial MVPA in this sample of frail or pre-frail older people (the baseline level of bouted MVPA was just 41 min per week). It should also be noted that accelerometers are designed to measure ambulatory physical activity rather than engagement in resistance exercise (which is mostly done while stationary). The 2·6-point change in the SF-36 physical component score was small (a clinically meaningful difference being cited as around 4 points),^38^, ^39^ as was the change in hand-grip strength (0·8 kg compared with a clinically important difference of 5·0 kg).^40^ This outcome shows that the functional benefits of the REACT programme were specific to the focus of the exercise intervention programme (lower limb mobility) and did not generalise to upper body physical functioning.

At 24 months, only changes in self-reported physical activity, muscle-strengthening exercise, and MAT-SF score (of the secondary outcomes) were sustained. Other differences found at 12 months were reduced (17 min per week unbouted MVPA and 1·5 points in the SF-36 physical component score) and fell below the level of significance, which might reflect deterioration of the effects on exercise behaviours over time, as well as insufficient statistical power to detect smaller differences.

The subgroup analyses suggested consistency of intervention effects across different subgroups of the population, including both sexes and people of different ages, education levels, and socioeconomic status.

To our knowledge, the REACT study is the largest translational trial done to date targeting long-term changes in lower limb physical function in older adults with mobility limitations. It was robustly designed, and had a low attrition rate (19% at 24 months) and good intervention adherence. Given the low dropout rate and the fact that the multiple imputation analysis showed no change in the results, the potential for bias due to attrition is low.

Although only 3% of those invited to take part were recruited, the REACT study invited everyone older than 65 years and then applied a two-stage screening process to remove non-eligible participants. From the study screening data, we estimate that more than 80% of those invited were likely to be ineligible due to having a SPPB score outside the target range or due to our other exclusion criteria. On this basis, the response rate among the eligible population was 17%. Furthermore, it is reassuring that the recruited sample was representative of the UK population older than 65 years, in terms of deprivation and ethnicity, except for an under-representation of south Asian older adults.^18^

The main limitation was that, similar to other studies of behavioural interventions, masking of the participants to study group was not possible, which introduces the possibility of social desirability bias in patient-reported measures. However, the primary outcome here consisted of a battery of physical tests assessed by independent observers with the data collectors masked to study group allocation. The secondary outcome analyses were exploratory, with no adjustment for multiple testing and should be interpreted accordingly. Due to the low numbers of participants from minority ethnic groups, the generalisability of the results to these populations needs to be established in future studies.

For older adults at risk of mobility limitations, programmes such as REACT could help sustain health and independence. Affordability is a key concern when commissioning public health services.

The REACT exercise intervention provides important evidence supporting WHO, US, and UK physical activity recommendations for multimodal exercise for adults older than 65 years.^41^, ^42^ The dose–response data support the idea that at least one multimodal exercise session per week (a fairly low level of commitment) could be sufficient to provide benefits on lower limb physical function. It is probable that the increased lower limb physical function in the intervention group followed engagement in the group sessions as suggested by the sensitivity analysis and the increased performance on regular muscle-strengthening exercise that was reported at all times. Mechanisms of behaviour change (eg, targeting the psychological needs of competence, autonomy, and relatedness), the quality of intervention delivery, and factors associated with intervention attendance and outcomes will be explored through a mixed-methods process evaluation, including a longitudinal qualitative study. These analyses will direct us to develop recommendations of good practice on how best to support older adults to maintain an active lifestyle after the intervention, for ongoing benefits on health and quality of life.

As recruitment to this trial was logistically challenging,^18^ further research is needed to identify a simple, sensitive, and specific screening assessment process to identify older adults who are likely to benefit from this type of intervention (ie, who have a SPPB score of 4–9). Such an assessment process will be useful both for future research and for implementation of the intervention in this population.

Future studies are also needed to examine the effectiveness of the REACT intervention in Black, Asian, and other minority ethnic populations, as well as to identify and address any barriers that might deter them from engaging with the programme. Further research is also needed to optimise the implementation of REACT at scale. For instance, it might be possible and synergistic to integrate the REACT intervention with existing mobility-related prevention and rehabilitation services. Contrary to the belief that older age comes with an inevitable decline in physical functioning, the REACT study shows that this decline can be slowed or even prevented with modest lifestyle changes.

## Data sharing

All source data used in this study are publicly available. The trial dataset can be accessed by contacting the corresponding author. All project documentation is available at https://www.fundingawards.nihr.ac.uk/award/13/164/51.

## Declaration of interests

We declare no competing interests.
