# Supplementary material for: Effect of a physical activity and behaviour maintenance programme on functional mobility decline in older adults: the REACT (Retirement in Action) randomised controlled trial
Source: Lancet Public Health. 2022 Mar 21;7(4):e316–26. doi: 10.1016/S2468-2667(22)00004-4 (PMC8967718; doi:10.1016/S2468-2667(22)00004-4)
Supplement: Supplementary appendix [file mmc1.pdf]

THE LANCET
Public Health

Supplementary appendix

This appendix formed part of the original submission and has been peer reviewed. We post it as supplied by the authors.

Supplement to: Stathi A, Greaves CJ, Thompson JL, et al. Effect of a physical activity and behaviour maintenance programme on functional mobility decline in older adults: the REACT (Retirement in Action) randomised controlled trial. *Lancet Public Health* 2022; published online March 21. https://doi.org/10.1016/S2468-2667(22)00004-4.

SUPPLEMENTARY FILE 1

Table S1: REACT measures and timepoints

Visit type	Scr	Scr	Fu	Fu	Fu
Visit code		SV1	F06	F12	F24
Visit number		1	2	3	4
Telephone call	1				
Activity/assessment Month	-0.5	0	6	12	24
**Form Name**					
Verbal consent	X				
Telephone screening (some elements of inclusion and exclusion criteria)	X				
Written informed consent		X			
Contact information update	X	X	X	X	X
Demographic data					
Age	X				
Gender	X				
Ethnicity	X				
*BMI (weight only at 12 months)		X		X	X
MoCA – Montreal Cognitive Assessment		X	X	X	X
Highest education level	X				
Index of Multiple Deprivation (IMD)	X				
Caring responsibilities		X			
Marital Status		X	X	X	X
Home ownership		X			
Number of chronic illnesses		X	X	X	X
SPPB battery		X	X	X	X
Accelerometry		X	X	X	X
Subjective Physical Activity (PASE questionnaire-10 item)		X	X	X	X
Muscle Strengthening Exercise Adherence Questionnaire		X	X	X	X
Dynometer (hand grip strength)		X	X	X	X
Ageing Well profile (6 items social well-being scale only at Bath/Bristol site)		X		X	X
Sleep Condition Indicator		X	X	X	X
Pain (Western Ontario and McMaster Universities Osteoarthritis Index (WOMAC)		X			X
Loneliness		X	X	X	X
Health-related quality of life (EQ-5D, SF-36)		X	X	X	X
Mobility Assessment Tool-Short Form (MAT-SF)		X	X	X	X
Cognitive function (UK Biobank Healthy Minds Questionnaire)		X	X	X	X
Falls Inventory (Number of falls and falls-related injuries in the last six months)		X	X	X	X
Health and Social Service Usage		X	X	X	X
Process Evaluation					
Session attendance (Intervention group only)			X	X	
Total contact time for each participant			X	X	
Physical activity related self-concept		X	X	X	
Perceived tension of maintaining current PA			X	X	X
Perceived tension of maintaining current exercise			X	X	X
Autonomy in relation to PA		X	X	X	X
Competence for PA		X	X	X	X
Relatedness for PA		X	X	X	X
Enjoyment of PA		X	X	X	X
Perceived intrinsic benefits of PA (social, physical and emotional)		X	X	X	X
Autonomy for strength-building exercise		X	X	X	X

Competence for strength-building exercise<br>Relatedness for strength-building exercise<br>Enjoyment of strength-building exercise<br>Perceived intrinsic benefits of strength-building exercise (social, physical and emotional)<br>Enjoyment of the REACT programme (Intervention group)<br>Credibility /identification with the session facilitators (Intervention group only)		X<br>X<br>X<br>X	X<br>X<br>X<br>X<br><br>X<br>X	X<br>X<br>X<br>X<br><br>X<br>X	X<br>X<br>X<br>X
(fMRI imaging substudy) MRI scan, detailed cognitive assessment and gait analysis		X	X	X	

*follow-up data collected to inform process analyses, **measured at each time point for screening/safety purposes only, ***to be reported elsewhere

Table S2: Primary and secondary outcomes at six months

	N	Control[a]	Intervention[a]	Estimated mean difference (95% CI)	p-value[b]
**Primary outcome**					
**SPPB battery (total score)**	305,354	8.07 (1.91)	8.74 (1.87)	0.68 (0.39, 0.96)	0.0009
**Secondary outcomes**					
**Accelerometry**					
MVPA (min/day): Time spent at >100mg in at least 10 min bouts	270, 314	5.68 (7.36)	6.56 (7.09)	0.86 (-0.37, 2.12)	0.17
MVPA (min/day): All time spent at >100mg	270,314	53.74 (18.17)	56.59 (22.42)	2.85 (-0.67, 6.37)	0.11
Sedentary time, excluding sleep (min/day)	261,304	810 (78.84)	809 (95.02)	-0.91 (-15.52, 13.71)	0.90
Breaks in sedentary time (N/day)	269,312	41.48 (9.51)	42.91 (9.71)	1.42 (-0.17, 3.02)	0.080
**Subjective PA (PASE)**	296,346	115.49 (58.21)	131.82 (61.76)	16.33 (6.78, 25.89)	0.0015
**Muscle-strengthening exercise (MSEQ)**	277, 320	3.22 (1.90)	3.93 (1.90)	0.70 (0.39, 1.01)	0.0004
**Hand grip strength (Kg),**	302,350	24.78 (3.93)	25.34 (4.09)	0.55 (-0.08, 1.18)	0.085
**Sleep Condition Indicator**	255,288	24.34 (5.74)	24.23 (5.45)	-0.16 (-1.05, 0.81)	0.81
**Loneliness**	301,345	111 (36.8%)	115 (33.3%)	-0.009 (-0.070, 0.052)	0.78
**SF-36**					
Physical Component	293,342	30.64 (8.68)	32.75 (8.42)	2.11 (0.73, 3.48)	0.0033
Mental Component	293,342	54.19 (7.46)	54.41 (6.88)	0.23 (-0.93, 1.38)	0.71
**EQ5D**	299,346	0.70 (0.15	0.68 (0.15)	0.026 (0.003, 0.05)	0.028
**MAT-SF**	297,345	50.00 (7.05)	51.40 (6.53)	1.40 (0.32, 2.48)	0.012
**UK Biobank Healthy Minds Questionnaire**					
Simple processing speed	287,333	811.79 (243.82)	821.25 (259.59)	9.45 (-32.48, 51.39)	0.66
Fluid intelligence	286,327	3.87 (1.42)	3.89 (1.32)	0.02 (-0.21, 0.25)	0.85
Executive function	240,270	62291.45 (39262.35)	60343.81 (39565.45)	-1947.64 (-8835.47, 4940.18)	0.58
Working memory 1	286,331	4.34 (1.21)	4.42 (1.13)	0.08 (-0.12, 0.29)	0.42
Working memory 2	286,330	13.59 (5.53)	13.83 (5.77)	0.24 (-0.70, 1.19)	0.61
**Episodic memory**	287,331	5.97 (4.98)	5.96 (4.59)	-0.00 (-0.80, 0.79)	0.99
**Falls Inventory**					
No. of falls in last 6 months	295,335	0.61 (0.93)	0.58 (1.02)	-0.03 (-0.18, 0.12)	0.71
Fall-related injury in last 6 months, *n* (%)	291,328	40 (13.7)	43 (13.1)	[c]0.64 (-4.96,6.35)	0.73

[a]mean (SD) unless otherwise stated
[b]adjusted for site, exercise group (within the intervention arm), age group, gender and baseline SPPB
[c]adjusted estimate and 95% CI for the between group percentage difference

Table S3: Primary and secondary outcomes at twelve months

	N	Control[a]	Intervention[a]	Estimated mean difference (95% CI)	p-value[b]
**Primary outcome**					
**SPPB battery (total score)**	303,346	7.85 (2.05)	8.62 (2.58)	0.77 (0.40, 1.14)	0.0010
**Secondary outcomes**					
**Accelerometry**					
MVPA (min/day): Time spent at >100mg in at least 10 min bouts	277,299	4.98 (5.51)	6.22 (6.20)	1.24 (0.22, 2.26)	0.018
MVPA (min/day): All time spent at >100mg	277,299	52.04 (15.92)	55.15 (19.67)	3.11 (-0.00, 6.23)	0.051
Sedentary time, excluding sleep (min/day)	274,300	790 (67.54)	789 (66.51)	-0.92 (-12.11, 10.27)	0.87
Breaks in sedentary time (N/day)	275,301	42.92 (9.62)	43.49 (10.93)	0.57 (-1.15, 2.28)	0.51
**Subjective PA (PASE)**	296,306	120.22 (49.34)	131.05 (46.23)	10.84 (3.18, 18.50)	0.0065
**Muscle-strengthening exercise (MSEQ)**	266, 315	3.32 (1.84)	3.98 (2.22)	0.67 (0.33, 1.00)	0.0006
**Hand grip strength[4] (Kg)**	297,340	24.42 (3.64)	25.23 (4.05)	0.81 (0.20, 1.43)	0.010
**Ageing Well Profile Social Wellbeing sub-scale**	284,322	24.34 (5.74)	24.23 (5.44)	-0.12 (-1.05, 0.81)	0.81
**Sleep Condition Indicator**	266,303	22.44 (5.28)	23.30 (4.89)	0.86 (-0.05, 1.77)	0.064
**Loneliness**	296,340	104 (35.1%)	111 (32.6)	-0.003 (-0.069, 0.064)	0.94
**SF-36**					
Physical Component	293,334	29.66 (8.53)	32.25 (8.23)	2.59 (1.22, 3.95)	0.0011
Mental Component	293,334	54.52 (7.52)	53.95 (7.81)	-0.57 (-1.82, 0.67)	0.36
**EQ5D**	293,337	0.70 (0.14)	0.69 (0.14)	0.018 (0.004, 0.041)	0.11
**MAT-SF**	292,328	49.25(7.51)	51.47 (7.79)	2.21 (1.00, 3.43)	0.0018
**UK Biobank Healthy Minds Questionnaire**					
Simple processing speed	257,297	829.66 (241.06)	842.97 (253.15)	13.31 (-30.01, 56.63)	0.54
Fluid intelligence	254,295	4.18 (1.47)	4.22 (1.54)	0.04 (-0.23, 0.31)	0.77
Executive function	204,244	66552.53 (44421.06)	62642.94 (44187.88)	-3909.59 (-12564.60, 4745.42)	0.37
Working memory 1	257,294	4.48 (1.35)	4.36 (1.52)	-0.13 (-0.38, 0.13)	0.32
Working memory 2	259,296	14.22 (5.68)	13.74 (5.83)	-0.49 (-1.50, 0.53)	0.34
Episodic memory	258,297	5.91 (4.87)	5.86 (4.45)	-0.05 (-0.88, 0.77)	0.90
**Falls inventory**					
No. of falls in last 6 months	300,330	0.73 (1.05)	0.70 (1.05)	-0.02 (-0.19, 0.14)	0.77
Fall-related injury in last 6 months, *n* (%)	297,326	51 (17.2)	57 (17.5)	0.3 (-5.92, 6.46)[c]	0.81

[a] mean (SD) unless otherwise stated
[b] adjusted for site, exercise group (within the intervention arm), age group, gender and baseline SPPB
[c] adjusted estimate and 95% CI for the between group percentage difference

Table S4: Primary outcome sensitivity analyses a) using multiple imputation b) without clustering by exercise group and c) using three population parameters to explore the impact of adherence

	Control SPPB total score (N, mean (SD)	Intervention SPPB total score (N, mean (SD)	Estimated mean difference (95% CI)	p-value
**Multiple imputation, N=777**	367, 7.50 (2.44)	410, 7.87 (2.93)	0.38 (0.02, 0.73)	0.040[a]
**With no clustering by exercise group[b], N=628**	294, 7.59 (2.61)	334, 8.7 (2.21)	0.49 (0.14, 0.84)	0.0062[c]
**Population 0 (whole sample), N=628**	294, 7.59 (2.61)	334, 8.08 (2.87)	0.49 (0.06, 0.92)	0.014[a]
**Population 1 (≥50% adherence), N=546**	294, 7.59 (2.20)	252, 8.23 (2.49)	0.64 (0.23, 1.05)	0.0028[a]
**Population 2 (≥75% adherence), N=471**	294, 7.64 (2.12)	177, 8.45 (2.28)	0.81 (0.38, 1.23)	0.0008[a]

[a] adjusted for site, exercise group (within the intervention arm), age group, gender and baseline SBBP.

[b]The intra-cluster correlation coefficient for SPPB scores relating to clustering by exercise group within the intervention arm was 0.02 (95%CI: 0.0085 to 0.129).

[c] adjusted for site, age group, gender and baseline SBBP

Table S5. Analysis of pre-specified subgroups. Sub-group variables added as covariates and interaction terms with study arm in main model.

Factor / Covariate	Coefficient (SE), P	Coefficient for interaction with study arm (SE), P[a]	Control Mean (SE)	Interventio n Mean (SE)
**Baseline SPPB**	[c]Chi2(1)=13.53 p<0.0001)	**(Chi^2(1)=0.06, p=0.82)**		
Baseline SPPB 4-7	0		6.20 (0.20)	6.71 (0.22)
Baseline SPPB 8-9	2.35 (0.19), 0.0003	0.094 (0.361), 0.794	8.59 (0.17)	9.03 (0.18)
**Education**	**(Chi^2(4)=2.50, p=0.64)**	Chi2(4) = 2.86 p=0.58		
Education level 1	0	0	7.41 .18 (0.44)	7.79 (0.45)
Education level 2	0.28 (0.35), 0.42	0.229 (0.690), 0.74	7.57 (0.20)	8.18 (0.23)
Education level 3	0.39 (0.36), 0.28	-0.317 (0.718), 0.66	8.00 (0.27)	8.06 (0.24)
Education level 4	0.15 (0.37), 0.70	0.303 (0.738) 0.68	7.39 (0.29)	8.08 (0.27)
Education level 5	-0.06 (0.46), 0.90	0.760 (0.926), 0.41	6.89 (0.54)	8.03 (0.42)
**Deprivation (**	**Chi^2(4)=17.79, p=0.014)**	Chi2(4)=1.77 p=0.78		
Deprivation level 1	0	0	7.14 (0.36)	7.68 (0.42)
Deprivation level 2	0.03 (0.34), 0.94	0.344 (0.670), 0.61	6.98 (0.30)	7.86 (0.29)

Factor / Covariate	Coefficient (SE), P	Coefficient for interaction with study arm (SE), P[a]	Control Mean (SE)	Interventio n Mean (SE)
Deprivation level 3	0.28 (0.34), 0.41	-0.343 (0.670), 0.60	7.60 (0.29)	7.80 (0.27)
Deprivation level 4	0.38 (0.35), 0.28	0.040 (0.668), 0.95	7.48 (0.29)	8.06 (0.27)
Deprivation level 5	1.04 (0.34), 0.0022	-0.211 (0.645), 0.74	8.28 (0.24)	8.61 (0.25)
**Age** (	**Chi^2**(1)=42.22, p<0.0001)	Chi2(1)= 0.01, p=0.93		
Age 65-74	0		8.35 (0.21)	8.78 (0.21)
Age 75+	-1.20 (0.19), 0.0006	-0.016 (0.368), 0.97	7.16 (0.17)	7.57 (0.18)
**Comorbidity (**	**Chi^2(1)=5.67, p=0.017**	Chi2(1)= 0.36, p=0.54		
Comorbidity No	0		7.86 (0.17)	8.17 (0.18)
Comorbidity Yes	-0.41 (0.18), 0.022	-0.283 (0.476), 0.55	7.19 (0.20)	7.97 (0.20)
**Gender (**	**Chi^2(1)=1.40, p=0.24)**	Chi2(1) = 0.53 p=0.46		
Gender: Male	0		7.54 (0.23)	7.85 (0.22)
Gender: Female	0.22 (0.19), 0.24	0.273 (0.375), 0.47	7.62 (0.16)	8.20 (0.17)
**Site (**	**Chi^2(2)=8.4, p=0.0150)**	Chi2(2) = 2.65, p=0.27		
Site: Bath / Bristol	0		7.18 (0.19)	7.95 (0.22)
Site: Birmingham	0.70 (0.25), 0.0070	-0.193 (0.497), 0.70	7.96 (0.27)	8.54 (0.29)
Site: Exeter	0.39 (0.23), 0.090	-0.711 (0.574), 0.12	7.88 (0.23)	7.94 (0.25)

Factor / Covariate	Coefficient (SE), P	Coefficient for interaction with study arm (SE), P[a]	Control Mean (SE)	Interventio n Mean (SE)
**Fallers (**	**Chi^2(1)=4.56, p=0.0328)**	Chi2(1)= 0.804 , p=0.37		
Fallers: Yes	0		7.48 (0.22)	7.76 (0.21)
Fallers: No	(0.18), 0.033	0.331 (0.365), 0.37	7.69 (0.16)	8.30 (0.17)
**Housing Type (**	**Chi^2(1)=7.32, p=0.0074)**	Chi2(1)=0.001 , p=0.96		
Home Owner	0		7.73 (0.14)	8.19 (0.14)
Other	-0.935 (0.26), 0.0075	0.012 (0.509), 0.98	6.79 (0.32)	7.27 (0.36)
**Other interventions[b] (**	**Chi^2(1)=0.09, p=0.76)**	Chi2(1)= 0.43, p=0.51		
24 Months: No	0		7.73 (0.25)	8.43 (0.31)
24 Months: Yes	0.08 (0.30), 0.78	-0.291 (0.634), 0.51	7.82 (0.16)	8.22 (0.15)

[a]Unstandardised coefficient for each factor in the regression model.
[b]Whether additional (non-REACT) physical activity interventions were undertaken at any point in the 24-month study period
[c] Overall comparison between groups using cross-tabulation (chi-squared) analysis applied to the estimated values for each level of the variable.

Multiple imputation specification

Multiple imputation was undertaken using multivariate normal regression in Stata version 15.0 and based on the creation of 10 imputed datasets. Imputation was undertaken primarily on the primary outcome, 24 month SPPB score. The baseline data was almost entirely complete with factors only missing between 1 and 11 of the 777 observations (Screening age (5 levels), Gender, Baseline SPPB score, Baseline BMI, Site, Education level at screening (5 levels), IMD quintile) and therefore no imputation of baseline values was undertaken. The 24 months SPPB score imputations were only calculated where a participants was known to be alive at the 24 month follow-up using multivariate normal regression. A combination of link functions were used for each variable imputed - normal for continuous variables, logit for binary and multinomial logit for data with more than 2 classes. There were 34 deaths during the trial (16, intervention, 18 control) and a total of SBBP total scores missing at 24 months of 150 observations from the potential total of 777 observations. This therefore gives a total non-death missing of 116 (18.5%) calculated in the imputation. Imputed values were checked to be within a plausible range. In order to obtain results consistent with the primary analysis the Kenward-Roger test was used in order to assess the effect of factors and covariates.

Table S6: Repeated measures mixed model analysis of the primary outcome (SBBP) measured at baseline, 6, 12 and 24 months post randomisation (adjusted for site, exercise group, age, gender and baseline SPPB score).

	Control Mean (SE)	Intervention Mean (SE)	Estimated mean difference[a] (95% CI)	p-value
**SPPB total score**				
Baseline	7.37 (0.12)	7.45 (0.28)		-
6 months	8.02 (0.13	8.75 (0.28)	0.65 (0.25, 1.03)	0.0016
12 months	7.83 (0.13)	8.65 (0.28)	0.74 (0.34, 1.13)	0.0010
24 months	7.54 (0.13)	8.11 (0.28)	0.48 (0.08, 0.88)	0.018

[a] Estimated group x time interaction effect on SPPB score.

Table S7. Details of unexpected serious adverse events

Study number	Allocation	Type	Related to intervention
AT1464	Intervention	Deceased - unknown cause	Not related
AT1564	Intervention	Stroke	Not related
AT1767	Intervention	Scheduled hip operation	Not related
AT1832	Intervention	Heart Attack	Not related
AT1905	Control	Deceased - unknown cause	Not related
AT1948	Intervention	Deceased - unknown cause	Not related
AT2171	Control	Deceased - unknown cause	Not related
AT2187	Intervention	Stomach ulcer	Not related
AT2275	Intervention	Mini stroke	Possible
AT2305	Intervention	Fall	Not related
AT2341	Intervention	Deceased - unknown cause	Not related
AT2414	Intervention	Deceased - Stroke	Not related
AT2486	Intervention	Deceased - unknown cause	Not related
AT2536	Intervention	Fall - Fractured hip	Related
AT2630	Control	Deceased - unknown cause	Not related
AT2648	Intervention	Deceased - unknown cause	Not related
AT2675	Intervention	Deceased - Frailty in old age	Not related
BT1231	Intervention	Deceased - Alzheimer’s disease	Not related
BT1348	Intervention	Cardiac event (non-fatal)	Not related
BT1585	Control	Cancer diagnosis	Not related
BT1636	Intervention	Severe nose bleeds	Not related
BT1704	Control	Deceased - cardiac event	not related
BT1753	Control	Deceased - suspected cardiac arrest	Not related
BT1770	Intervention	Cancer diagnosis	Not related
BT1785	Intervention	Heart Attack	Not related
BT1979	Control	Fall - broken shoulder	Not related

BT2002	Control	Bowel Cancer diagnosis	Not related
ET1036	Control	Fall - injured arm and head (hospitalised). Diagnosed with pneumonia and kidney issues.	Not related
ET1044	Control	Stroke	Not related
ET1073	Intervention	Diarrhoea, dehydration and rheumatoid arthritis	Not related
ET1295	Control	Stomach pains, a hernia, and memory deterioration	Not related
ET1392	Intervention	Sciatica which aggravated MS	Not related
ET1392	Intervention	Spinal surgery, fractured sacrum due to surgery	Not related
ET1393	Control	Fall - fractured head of femur, led to half hip preplacement	Not related
ET1425	Intervention	Non-Hogkins lymphoma	Not related
ET1439	Intervention	Spine compression	Not related
ET1506	Intervention	Elective surgery resulting in sepsis and pneumonia	Not related
ET1565	Intervention	Fall - socket of femur break. Hip replacement needed.	Not related
ET1603	Control	Bad reaction to flu injection needing cardioversion	Not related
ET1689	Control	Sepsis	Not related
ET1717	Intervention	Pneumonia	Not related
ET1752	Control	Deceased - pneumonia, emphysema and lung cancer	Not related
ET1754	Intervention	Terminal cancer diagnosis	Not related
ET1804	Control	Asthma attack (hospitalised), followed by fall resulting in brain bleed.	Not related
ET1844	Control	Collapsed at home - suspected bleeding ulcer	Not related
ET1866	Intervention	Deceased - colon cancer	Not related

ET1953	Intervention	Knee injury	Not related
ET1959	Control	Fall - double fracture and dislocation of left ankle	Not related
ET1969	Intervention	Fall - cracked ribs	Not related
ET1977	Intervention	Fall - broken hip	Not related
ET1999	Intervention	Elective surgery - swollen testicles	Not related
ET2017	Control	TIA (hospitalised)	Not related
ET2024	Control	Fall - broken shoulder	Not related
ET2024	Control	Dementia diagnosis	Not related
ET2035	Control	Two stents fitted and aortic valve replaced	Not related
ET2036	Intervention	Hernia (surgery performed)	Not related
ET2045	Intervention	Chest pains (high troponin levels), contracted flu in hospital	Not related
ET2067	Intervention	Pneumonia (hospitalised). Minor heart attack while hospitalised.	Not related
ET2067	Intervention	Pneumonia, second bout (hospitalised)	Not related
ET2069	Control	Fall - fractured Humerus	Not related
ET2069	Control	Planned knee replacement	Not related
ET2081	Intervention	TIA	Not related
ET2092	Control	Sepsis	Not related
ET2117	Intervention	Bleeding (details unknown)	Not related
ET2128	Control	Deceased - unknown cause	Not related
ET1281	Intervention	Cardiac issues	Not related
ET2191	Intervention	Triple fusion of ankle joint	Not related
ET2205	Intervention	Cellulitis	Not related
ET2255	Intervention	Stroke	Not related
ET2255	Intervention	Fall (hospitalised)	Not related
ET2290	Intervention	Stroke	Not related
ET2309	Intervention	Lung cancer	Not related

ET2374	Control	Accident - fractured hip resulting in replacement	Not related
ET2377	Control	Stroke	Not related
ET2402	Control	Fall - haematoma on leg	Not related
ET2401	Intervention	Pneumonia (hospitalised). Treatment caused AF and bleed to abdomen.	Not related
ET2416	Intervention	Planned knee replacement	Not related
ET2416	Intervention	Problems with knee replacement	Not related
ET2425	Intervention	Terminal bone cancer	Not related
ET2449	Intervention	Deceased - heart attack and possible appendicitis	Not related
ET2455	Intervention	Heart attack - three stents fitted	Not related
ET2472	Control	Chest pains - stents fitted	Not related
ET2481	Control	TIA	Not related
ET2511	Intervention	Big toe removed	Not related
ET2528	Control	Cardiac 'episode' (hospitalised)	Not related
ET2357	Intervention	Hospitalisation (pneumonia)	Not related
ET2588	Intervention	COPD (hospitalised). On palliative care.	Not related
ET2709	Intervention	Fall - broke left femur	Not related
ET2710	Intervention	Sepsis, pneumonia and pleurisy	Not related
ET2710	Intervention	Deceased - Ischemic heart disease, COPD and diabetes.	Not related
ET2716	Control	Gall stone in bowel	Not related
ET2733	Control	Fall (hospitalised) - pacemaker fitted	Not related
ET2733	Intervention	Hematoma (hospitalised)	Not related

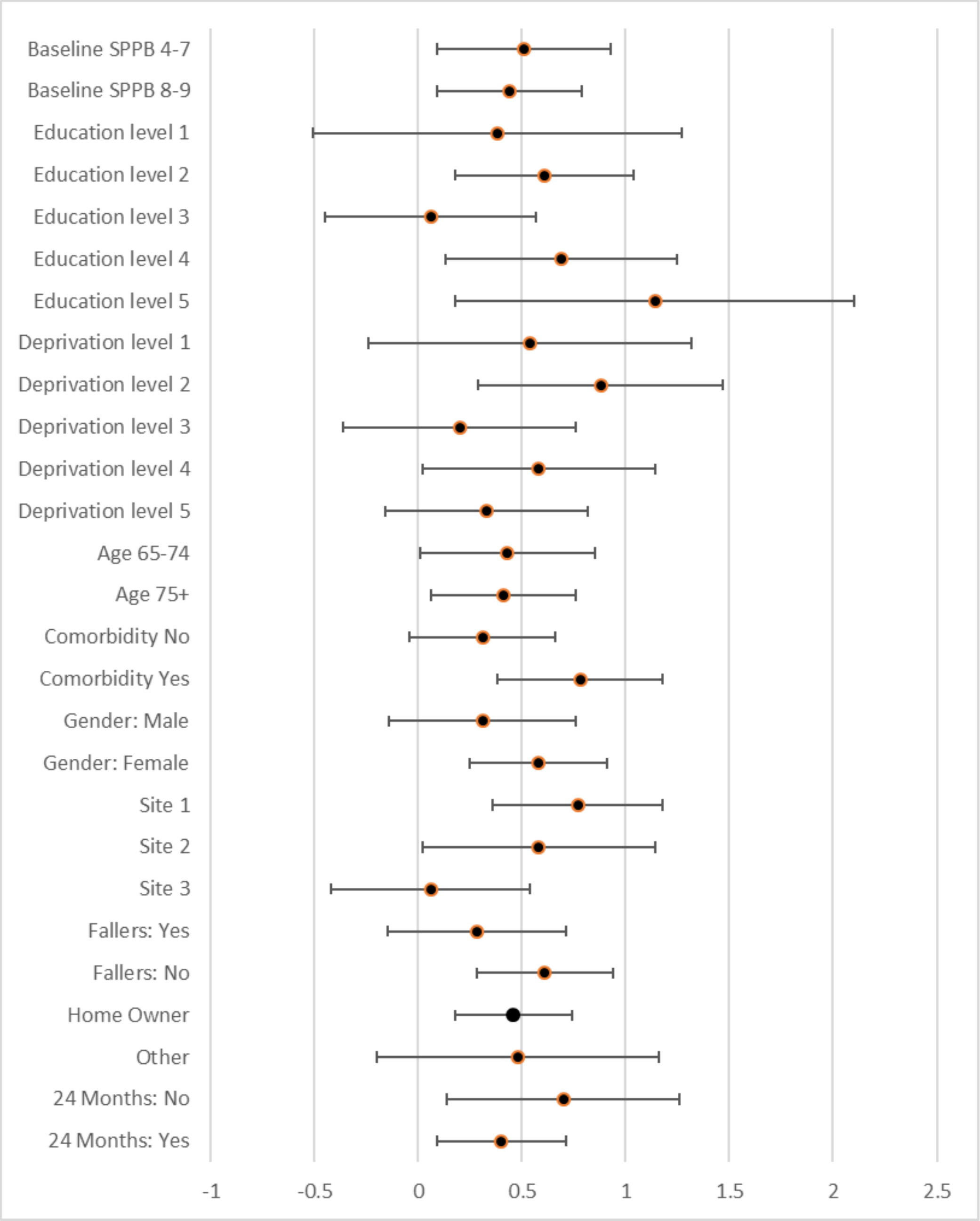

Figure S1: Forest Plot

SUPPLEMENTARY FILE 2

Intervention costs

Referral and coordination: REACT is a group-based intervention which necessarily requires a minimum number of eligible people to run. It is expected that these people will be identified by general practitioners and referred to a social prescribing link worker who will coordinate REACT groups for the local primary care network. Identifying suitable people is assumed not to have an opportunity cost in terms of GP time, but making a referral is expected to take some time, although costs of referral have generally been omitted from prior economic evaluations of physical activity referral schemes references. Our base case assumption is that there will be no incremental GP time cost associated with referral, as the time taken to make a referral is not expected to be significant or notably different from inputs in the usual care are such as GP advice to increase physical activity, for which no costs have been included in the analysis. We include 30 minutes of time for a social prescribing link worker per patient, whose job is to ensure that the patient is appropriate for referral, briefly describe the REACT intervention and then refer the patient to a REACT coordinator (see below). REACT study investigators have estimated that to screen an individual for the REACT study took 30 minutes.[1] As a sensitivity analysis, we replace these costs with the estimated recruitment costs for the REACT study, which included screening GP databases to identify suitable individuals, mailing invitations, a PR campaign and other recruitment methods, at an estimated total cost of £81,597 to recruit 777 participants (£105.02 per participant recruited). [1]

The REACT coordinator will perform activities such as:

- Identifying appropriate venues and making bookings
- Recruiting personal trainers (as necessary)
- Ensuring that personal trainers are trained appropriately to deliver REACT and are intermittently supervised for intervention fidelity
- Maintaining a waiting list
- Administering related payments (to venues and trainers and other expenses)
- Sending schedules and reminders to participants

We assume that these activities will require 36 hours of coordinator time (approximately one working week) per REACT group based on discussion with the investigators. The coordinator may be employed by the provider or employed by the funder.

Table S61. Cost of the REACT intervention

Item	Resource use per group	Unit cost (£)	Cost per group (£)	Cost per participant (£)
Link worker	30-minute pp*	33.83	257.48	16.92
Coordinator	36-hour per group	33.83	1217.88	80.01
Introductory sessions	45-minute pp*	29.43	335.99	22.07
Equipment				
- Pedometers	One pp*	9.95	151.46	9.95
- Other	Ankle weights and therabands, assumes reuse and sharing between groups	29.16	29.16	1.92
**REACT session leader**				
- Preparation	30-minute ps**	29.43	941.76	61.87
- Travel time	30 minutes ps**	29.43	941.76	61.87
- Delivery	90 minutes ps**	29.43	2825.28	185.60
Consumables				
- Refreshments	Approx. 1 per participant per session	1.00	621.32	40.82
- Printed materials	One set pp*	2.00	30.44	2.00
Venue hire	90-minute ps**	17.32	1662.72	109.23
Training session leaders				
- Training leaders	One leader to four trainees	33.83	22.20	1.46

- REACT session leaders time	1.5 days for every four programmes delivered	29.43	77.25	5.08
Training venue	As above	29.43	77.25	5.07
- Training manual	One per REACT trainer	12.00	3.00	0.20
**Total**			**9465.69**	**621.83**

pp* = per participant; ps** = per session.

REACT session leader: The NHS does not in general employ sports and fitness instructors to deliver an intervention like REACT, so the services of such instructors would need to be purchased from the private or independent sector. The mean hourly salary for an employed fitness instructor was estimated to be £10.63.[2] However, over half of fitness instructors are self-employed[3] and the hourly charge for these is not well estimated by national statistics. In the REACT study all trainers were qualified to at least level 3 (whereas many fitness instructors may only be qualified to level 2). One REACT study provider reported a base salary cost of £15.50 per hour, and one freelance trainer charged £26 per hour. A recent economic evaluation of individual health trainers[4] estimated the cost of a health trainer using the Agenda for Change band 4 cost information from the PSSRU, an hourly cost of £28 (revised to £29.43 in the most recent version of PSSRU unit costs)[5]. The base annual salary for band 4 community-based scientific and professional staff is £22,256, which spread over 1,618 working hours per year (as assumed for Band 4 workers by PSSRU) corresponds to an hourly gross salary of £13.76. We assume that the cost of REACT trainers will be £29.43 per hour and this should not be highly sensitive to whether REACT trainers are employed or freelance. The total cost of £29.43 per hour includes salary oncosts (23% of base salary), overheads (77% of base salary) and capital costs (14% of base salary). We therefore assume that this includes the cost of any supervision required, a suitable location to meet REACT participants and perform any administrative duties, and any travel expenses. In sensitivity analyses we vary the cost by ± 20%.

Venue hire: The NHS does not (in general) have facilities to accommodate an intervention like REACT, which requires a hall or large room suitable for group exercise with up to 15 participants. It is expected that if the REACT intervention were to be rolled out it would require hiring of a venue, which might be maintained by the private sector, the charity/non-profit sector or by a public body (e.g., local authority community centres, schools). The costs of hiring such a venue will likely vary significantly depending on geographical location (particularly land value), type of space and facilities (determines other demands on space) and in which sector it is located. The REACT intervention assumes operation during off-peak hours for health centres. The cost of venue hire was reported by the investigators as £15 to £25 per hour for some sites, £32.50 per session for a multi-site provider, while for a number of sites an overall cost was reported including costs for staff and refreshments so no venue cost could be identified. The cost of venue hire was estimated to be £17.32 per hour following a survey of randomly chosen locations in England covering all ten deciles of index of multiple deprivation.

To attempt to identify a representative sample of venue hire costs from outside the REACT trial, we randomly sampled ten lower-layer super output areas (LSOAs) in England, one from each decile of index of multiple deprivation[6]. LSOAs have similar populations (approximately 1,500 residents). For each LSOA we took the central latitude and longitude and attempted to locate a suitable venue for the REACT intervention and identify a likely cost based on publicly available information or by telephone/email contact. The retrieved prices ranged from £12 to £25 per hour, with mean £17.32 per hour, which is used as the base case cost per hour of venue hire. In sensitivity analyses we vary the cost from £15 per hour to £25 per hour.

One-to-one introductory sessions: We assume that the 45 minute introductory sessions are attended by all individuals allocated to the REACT intervention. As this may be an overestimate (some may not attend the introductory session and it is unlikely the opportunity cost of a non-attendance is equal to the opportunity cost of a session), we do not include other preparation or travel costs for these sessions. In a sensitivity analysis we include an additional 30 minutes of REACT trainer time for travel and/or preparation.

Cost per session: The cost per session of REACT is estimated from the REACT trainer time (preparation, travel and delivery), REACT trainer travel expenses, venue hire and consumables. The

nominal session lengths are 80 minutes (41 sessions) and 105 minutes (23 sessions), giving a mean nominal session length of 89 minutes. A number of factors may result in the costs associated with the sessions not matching the nominal lengths of the sessions, such as the need for setup and tidying up at the start and end of the session, or venues being only bookable in blocks which are multiples of 15, 20, 30 or 60 minutes. Trainers were requested to note the start and end times of each session, and this provides empirical data on the length of sessions (719 sessions provided these data out of a maximum 1728). The data suggest that a significant proportion of 80 minute sessions were shortened to 75 minutes or extended to 90 minutes, and that some 105 minute sessions may have been shortened to 90 minutes. The mean session length was 86.3 minutes. We estimate the cost per session by assuming each session requires 90 minutes of REACT trainer time and venue hire. In a sensitivity analysis we vary this from 80 minutes to 105 minutes.

Trainer preparation time was not universally recorded, with 37% of sessions not having a recorded trainer preparation time. Trainers were heterogeneous in their recording of preparation time; for example, the trainer with the most recorded sessions (189) recorded preparation time in 96% (181) of those sessions, while the trainer with the second-most recorded sessions (160) did not record preparation time at all. Trainers were also heterogeneous in the amount of time they spent preparing. One multisite provider invoiced for 158 hours for delivering the 64 session REACT programme; if we assume 90 minutes per session and 45 minutes for 15 participants of 1:1 introductory sessions, this leaves ~51 hours (~48 minutes per session) for preparation. In the base case we assume 30 minutes of preparation per session. In sensitivity analyses we vary this from 10 minutes to 90 minutes.

Trainer travel time was only recorded in 139 of 737 (18.9%) of session records, with a mean travel time of 22.2 minutes in those sessions. One multisite provider invoiced for 45 minutes travel per session. In the base case we assume 30 minutes travel per session (15 minutes each way). In a sensitivity analysis we remove travel time, to represent the cost if staff are already on site.

Consumables per session were assumed to be limited to refreshments, and to cost £1 per attendee per session. As attendance at the first session is a good estimate of average attendance, we assumed that refreshments would be ordered for the full class for the first session (mean 14.63), and thereafter would be ordered for the number attending the first session (mean 9.63).

Equipment: Each REACT participant was provided with a pedometer costing £9.95. Other equipment was provided to sites to share between groups. Each site was provided with eight sets of ankle weights (at a cost of £119.92 per site) and therabands (at a cost of £16.35 per site). We assumed each site would use the equipment for five groups before replacing it. Additionally the cost was amortised over five years with a discount rate of 3.5% per year, meaning the total cost attributed to each group was 21.4% of the replacement cost (i.e., £29.16) rather than 20%.

Printed materials: The REACT intervention does not include a significant number of printed materials for participants. These were estimated to be less than £2 per participant by the investigators. We assume a cost per participant of £2.

Group size: The REACT intervention is designed for groups up to 15, but most groups in the study included at least one non-starter, and a number of groups included 16 or 17 invitees. There were a total 411 randomised and these were allocated to 27 groups, so the average group size was 15.2. In sensitivity analyses we vary the group size from 12 to 17.

References

1. Withall J, Greaves CJ, Thompson JL, de Koning JL, Bollen JC, Moorlock SJ, *et al.* The Tribulations of Trials: Lessons Learnt Recruiting 777 Older Adults Into REtirement in ACTion (REACT), a Trial of a Community, Group-Based Active Aging Intervention Targeting Mobility Disability. *The Journals of Gerontology: Series A* 2020; 10.1093/gerona/glaa051. http://dx.doi.org/10.1093/gerona/glaa051

2. Office for National Statistics. *Annual Survey of Hours and Earnings (2019 provisional) - Earnings and hours worked, occupation by four-digit SOC: ASHE Table 14*. 2019. URL: https://www.ons.gov.uk/employmentandlabourmarket/peopleinwork/earningsandworkinghours/datasets/occupation4digitsoc2010ashetable14 (Accessed February 7, 2020).
3. Office for National Statistics. *EMP04: Employment by occupation (April to June 2018)*. 2018. URL: https://www.ons.gov.uk/employmentandlabourmarket/peopleinwork/employmentandemployeetypes/datasets/employmentbyoccupationemp04 (Accessed February 7, 2020).
4. Callaghan L, Thompson TP, Creanor S, Quinn C, Senior J, Green C*, et al.* Individual health trainers to support health and well-being for people under community supervision in the criminal justice system: the STRENGTHEN pilot RCT. 2019;**7**:20. http://dx.doi.org/10.3310/phr07200
5. Curtis L, Burns A. Unit Costs of Health and Social Care 2019. Canterbury: Personal Social Services Research Unit, University of Kent; 2018.
6. Department for Communities and Local Government. *English Indices of Deprivation 2019 - LSOA Level*. 2019. URL: http://opendatacommunities.org/data/societal-wellbeing/imd2019/indices (Accessed February 5, 2020).

SUPPLEMENTARY FILE 3

CONSORT 2010 checklist of information to include when reporting a randomised trial*

Section/Topic	Item No	Checklist item	Reported on page No
**Title and abstract**			
	1a	Identification as a randomised trial in the title	1
	1b	Structured summary of trial design, methods, results, and conclusions (for specific guidance see CONSORT for abstracts)	2
**Introduction**			
Background and objectives	2a	Scientific background and explanation of rationale	3
	2b	Specific objectives or hypotheses	4
**Methods**			
Trial design	3a	Description of trial design (such as parallel, factorial) including allocation ratio	4-6
	3b	Important changes to methods after trial commencement (such as eligibility criteria), with reasons	5-See attached Statistical Analysis Plan
Participants	4a	Eligibility criteria for participants	4
	4b	Settings and locations where the data were collected	7
Interventions	5	The interventions for each group with sufficient details to allow replication, including how and when they were actually administered	6-7
Outcomes	6a	Completely defined pre-specified primary and secondary outcome measures, including how and when they were assessed	8, Table A2
	6b	Any changes to trial outcomes after the trial commenced, with reasons	N/A
Sample size	7a	How sample size was determined	9/10
	7b	When applicable, explanation of any interim analyses and stopping guidelines	See attached Protocol

Randomisation:			
Sequence generation	8a	Method used to generate the random allocation sequence	5
	8b	Type of randomisation; details of any restriction (such as blocking and block size)	5
Allocation concealment mechanism	9	Mechanism used to implement the random allocation sequence (such as sequentially numbered containers), describing any steps taken to conceal the sequence until interventions were assigned	5
Implementation	10	Who generated the random allocation sequence, who enrolled participants, and who assigned participants to interventions	5
Blinding	11a	If done, who was blinded after assignment to interventions (for example, participants, care providers, those assessing outcomes) and how	5
	11b	If relevant, description of the similarity of interventions	7
Statistical methods	12a	Statistical methods used to compare groups for primary and secondary outcomes	9-10
	12b	Methods for additional analyses, such as subgroup analyses and adjusted analyses	10-11
**Results**			
Participant flow (a diagram is strongly recommended)	13a	For each group, the numbers of participants who were randomly assigned, received intended treatment, and were analysed for the primary outcome	Figure 1
	13b	For each group, losses and exclusions after randomisation, together with reasons	10 and Figure 1
Recruitment	14a	Dates defining the periods of recruitment and follow-up	13
	14b	Why the trial ended or was stopped	N/A
Baseline data	15	A table showing baseline demographic and clinical characteristics for each group	Table 1
Numbers analysed	16	For each group, number of participants (denominator) included in each analysis and whether the analysis was by original assigned groups	12
Outcomes and estimation	17a	For each primary and secondary outcome, results for each group, and the estimated effect size and its precision (such as 95% confidence interval)	13-14 Table 2
	17b	For binary outcomes, presentation of both absolute and relative effect sizes is recommended	N/A
Ancillary analyses	18	Results of any other analyses performed, including subgroup analyses and adjusted analyses, distinguishing pre-specified from exploratory	13, 14,
Harms	19	All important harms or unintended effects in each group (for specific guidance see CONSORT for harms)	14
**Discussion**			
Limitations	20	Trial limitations, addressing sources of potential bias, imprecision, and, if relevant, multiplicity of analyses	17

Generalisability	21	Generalisability (external validity, applicability) of the trial findings	17
Interpretation	22	Interpretation consistent with results, balancing benefits and harms, and considering other relevant evidence	16-23
**Other information**			
Registration	23	Registration number and name of trial registry	9
Protocol	24	Where the full trial protocol can be accessed, if available	5 (Ref 14) https://doi.org/10.1186/s13063-018-2603-x
Funding	25	Sources of funding and other support (such as supply of drugs), role of funders	2,11

SUPPLEMENTARY FILE 4

TIDieR
Template for Intervention Description and Replication

The TIDieR (Template for Intervention Description and Replication) Checklist*:

Information to include when describing an intervention and the location of the information

Item number	Item	Where located ** Primary paper (page or appendix number)	Other † (details)
	**BRIEF NAME**		
**1.**	Provide the name or a phrase that describes the intervention.	____1________	______________
	**WHY**		
**2.**	Describe any rationale, theory, or goal of the elements essential to the intervention.	___3-4 _____	______________
	**WHAT**		
**3.**	Materials: Describe any physical or informational materials used in the intervention, including those provided to participants or used in intervention delivery or in training of intervention providers. Provide information on where the materials can be accessed (e.g. online appendix, URL).	_____________	__https://www.thereactstudy.com/___________
**4.**	Procedures: Describe each of the procedures, activities, and/or processes used in the intervention, including any enabling or support activities.	___5-6_______	______________
	**WHO PROVIDED**		
**5.**	For each category of intervention provider (e.g. psychologist, nursing assistant), describe their expertise, background and any specific training given.	____6,_______	______________
	**HOW**		

**6.**	Describe the modes of delivery (e.g. face-to-face or by some other mechanism, such as internet or telephone) of the intervention and whether it was provided individually or in a group.	6-7	
	**WHERE**		
**7.**	Describe the type(s) of location(s) where the intervention occurred, including any necessary infrastructure or relevant features.	6	
	**WHEN and HOW MUCH**		
**8.**	Describe the number of times the intervention was delivered and over what period of time including the number of sessions, their schedule, and their duration, intensity or dose.	6-7	
	**TAILORING**		
**9.**	If the intervention was planned to be personalised, titrated or adapted, then describe what, why, when, and how.	6	
	**MODIFICATIONS**		
**10.‡**	If the intervention was modified during the course of the study, describe the changes (what, why, when, and how).	N/A	
	**HOW WELL**		
**11.**	Planned: If intervention adherence or fidelity was assessed, describe how and by whom, and if any strategies were used to maintain or improve fidelity, describe them.	9	REACT study protocol: https://doi.org/10.1186/s13063-018-2603-x
**12.‡**	Actual: If intervention adherence or fidelity was assessed, describe the extent to which the intervention was delivered as planned.	15	

Supplementary File 5

(REtirement in ACTion)
Questionnaire

We need your help to make our study a success.
Remember that…

- **We want to know what you think about things, and understand more about your feelings and opinions**
- **There are no right or wrong answers – please just be as honest as you feel you can be**
- **We suggest you go with your 'gut reaction' and don't over-think answers**
- **Everything you tell us will be kept strictly confidential (secret) and you're free to decline answering any questions you're not comfortable with**

For Researcher Use

Measures 1 ❒ Measures 2 ❒ Measures 3 ❒ Measures 4 ❒

Date: ____________________ Researcher initials: _________

Was another person present/contributing during the questionnaire delivery?

Yes/No **If ‘yes,’ who: _____________________**

Data entry researcher

ID CODE: ___________________ Data entered: __________ Initials: _______

Ageing Well Profile

In this section, we're interested in your well-being during the **last month**. Choose the box (✔) that best describes your feelings and thoughts about your health.

About your life…

<u>During the last month</u>:	Not really true for me		Sort of true for me		Really true for me
I have managed to sort out all my needs by myself.	1 ☐	2 ☐	3 ☐	4 ☐	5 ☐
In everyday life, I have <u>not</u> needed to rely on others.	1 ☐	2 ☐	3 ☐	4 ☐	5 ☐
I have been able to take good care of myself.	1 ☐	2 ☐	3 ☐	4 ☐	5 ☐
My life has given me a sense of accomplishment.	1 ☐	2 ☐	3 ☐	4 ☐	5 ☐
I have felt some good changes in myself.	1 ☐	2 ☐	3 ☐	4 ☐	5 ☐
My life has been really interesting.	1 ☐	2 ☐	3 ☐	4 ☐	5 ☐
I have felt that I have improved myself.	1 ☐	2 ☐	3 ☐	4 ☐	5 ☐

About your health and fitness…

**During the last month:**	**Not really true for me**		**Sort of true for me**		**Really true for me**
I have usually woken up fresh and rested	1 ☐	2 ☐	3 ☐	4 ☐	5 ☐
My body has felt strong enough to do what I wanted to do	1 ☐	2 ☐	3 ☐	4 ☐	5 ☐
On a day to day basis I have felt lively and healthy	1 ☐	2 ☐	3 ☐	4 ☐	5 ☐
I have not had many pains or much discomfort	1 ☐	2 ☐	3 ☐	4 ☐	5 ☐
My body has felt old and has limited what I could do	1 ☐	2 ☐	3 ☐	4 ☐	5 ☐
I have been able to keep going for longer than most people my age	1 ☐	2 ☐	3 ☐	4 ☐	5 ☐

About your feelings…

**During the last month:**	**Not really true for me**		**Sort of true for me**		**Really true for me**
I have had no doubts about who I am or my worth	1 ☐	2 ☐	3 ☐	4 ☐	5 ☐
I have rarely felt confused	1 ☐	2 ☐	3 ☐	4 ☐	5 ☐
I have not worried much about my life	1 ☐	2 ☐	3 ☐	4 ☐	5 ☐
I have been able to concentrate well when I wanted	1 ☐	2 ☐	3 ☐	4 ☐	5 ☐
I have had complete confidence in myself and my decisions	1 ☐	2 ☐	3 ☐	4 ☐	5 ☐
I have felt contented and happy with myself	1 ☐	2 ☐	3 ☐	4 ☐	5 ☐
I have been in a good mood more often than not	1 ☐	2 ☐	3 ☐	4 ☐	5 ☐

About your social life…

**During the last month:**	**Not really true for me**		**Sort of true for me**		**Really true for me**
I have had plenty of people available to share my problems with	1 ☐	2 ☐	3 ☐	4 ☐	5 ☐
I have felt that people have enjoyed my company	1 ☐	2 ☐	3 ☐	4 ☐	5 ☐
I have rarely felt lonely or isolated	1 ☐	2 ☐	3 ☐	4 ☐	5 ☐
I have had lots of friends who I wanted to spend time with	1 ☐	2 ☐	3 ☐	4 ☐	5 ☐
I have spent a lot of my time with friends and acquaintances	1 ☐	2 ☐	3 ☐	4 ☐	5 ☐
My social life has been as good, as I would have liked	1 ☐	2 ☐	3 ☐	4 ☐	5 ☐
I have been able to give support and friendship to other people	1 ☐	2 ☐	3 ☐	4 ☐	5 ☐

The Sleep Condition Indicator

Please circle your answer

Item	Score				
	**4**	**3**	**2**	**1**	**0**
**Thinking about a typical night in the last month…**					
1. … how long does it take you to fall asleep?	0-15 min	16-30 min	31-45 min	46-60 min	≥61 min
2. …if you then wake up during the night … how long are you awake for in total? (add all the wakenings up)	0-15 min	16-30 min	31-45 min	46-60 min	≥61 min
3. …how many nights a week do you have a problem with your sleep?	0-1	2	3	4	5-7
4. … how would you rate your sleep quality?	Very good	Good	Average	Poor	Very poor
**Thinking about the past month, to what extent has poor sleep…**					
5. …affected your mood, energy, or relationships?	Not at all	A little	Somewhat	Much	Very much
6. …affected your concentration, productivity, or ability to stay awake?	Not at all	A little	Somewhat	Much	Very much
7. …troubled you in general?	Not at all	A little	Somewhat	Much	Very much
**Finally …**					
8. …how long have you had a problem with your sleep?	I don't have a problem/ Less than 1 month	1-2 months	3-6 months	7-12 months	More than 1 year

Hip, Knee, Ankle Joint Pain

The following questions will asked in the following format and you should give your answers by putting an “X” in one of the boxes.

NOTE: 1. If you put your “X” in the left-hand box, i.e.

None	Mild	Moderate	Severe	Extreme
☒	☐	☐	☐	☐

Then you are indicating that you have no pain.

2. If you put your “X” in the right-hand box, i.e.

None	Mild	Moderate	Severe	Extreme
☐	☐	☐	☐	☒

Then you are indicating that your pain is extreme.

The following question concern the amount of pain you have experienced in your hip, knee or ankle joints. For each situation please enter the amount of pain experienced during the last week. (Please mark your answers with an ☒)

QUESTION: How much pain do you have?

1. Walking on a flat surface.

None	Mild	Moderate	Severe	Extreme
☐	☐	☐	☐	☐

2. Going up or down stairs.

None	Mild	Moderate	Severe	Extreme
☐	☐	☐	☐	☐

3. At night while in bed.

None	Mild	Moderate	Severe	Extreme
☐	☐	☐	☐	☐

4. Sitting or lying.

None	Mild	Moderate	Severe	Extreme
☐	☐	☐	☐	☐

5. Standing upright.

None	Mild	Moderate	Severe	Extreme
☐	☐	☐	☐	☐

EQ-5D-5L

Under each heading, please tick the ONE (✔) that best describes your health TODAY.

MOBILITY

I have no problems in walking about	☐
I have slight problems in walking about	☐
I have moderate problems in walking about	☐
I have severe problems in walking about	☐
I am unable to walk about	☐

SELF-CARE

I have no problems washing or dressing myself	☐
I have slight problems washing or dressing myself	☐
I have moderate problems washing or dressing myself	☐
I have severe problems washing or dressing myself	☐
I am unable to wash or dress myself	☐

USUAL ACTIVITIES *(e.g. work, study, housework, family or leisure activities)*

I have no problems doing my usual activities	☐
I have slight problems doing my usual activities	☐
I have moderate problems doing my usual activities	☐
I have severe problems doing my usual activities	☐
I am unable to do my usual activities	☐

PAIN / DISCOMFORT

I have no pain or discomfort	☐
I have slight pain or discomfort	☐
I have moderate pain or discomfort	☐
I have severe pain or discomfort	☐
I have extreme pain or discomfort	☐

ANXIETY / DEPRESSION

I am not anxious or depressed	☐
I am slightly anxious or depressed	☐
I am moderately anxious or depressed	☐
I am severely anxious or depressed	☐
I am extremely anxious or depressed	☐

- We would like to know how good or bad your health is TODAY.
- This scale is numbered from 0 to 100.
- 100 means the <u>best</u> health you can imagine. 0 means the <u>worst</u> health you can imagine.
- Mark an X on the scale to indicate how your health is TODAY.
- **Now, please write the number you marked on the scale in the box below.**

YOUR HEALTH TODAY = ☐

The best health you can imagine

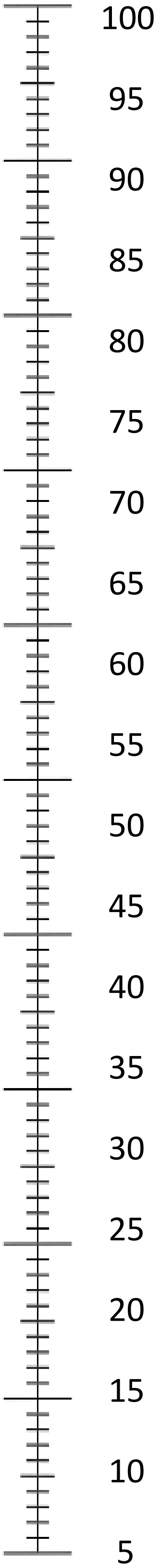

The worst health you can imagine

SF-36

1. In general, would you say your health is:

Excellent	Very good	Good	Fair	Poor
1	2	3	4	5

2. Compared to one year ago, how would you rate your health in general now:

Much better	Somewhat better	About the same	Somewhat worse	Much worse
1	2	3	4	5

The following items are about activities you might do during a typical day. Does **your health now limit you** in these activities? If so, how much?

(Circle One Number on Each Line)

	Yes, limited a lot	Yes, limited a little	No, not limited at all
3. **Vigorous activities**, such as running, lifting heavy objects, participating in strenuous sports	[1]	[2]	[3]
4. **Moderate activities**, such as moving a table, pushing a vaccuum cleaner, bowling, or playing golf	[1]	[2]	[3]
5. Lifting or carrying groceries	[1]	[2]	[3]
6. Climbing **several** flights or stairs	[1]	[2]	[3]
7. Climbing **one** flight of stairs	[1]	[2]	[3]
8. Bending, kneeling, or stooping	[1]	[2]	[3]
9. Walking **more than a mile**	[1]	[2]	[3]
10. Walking **several hundred yards**	[1]	[2]	[3]

11. Walking **one hundred yards**	[1]	[2]	[3]
12. Bathing or dressing yourself	[1]	[2]	[3]

During the **past 4 weeks**, have you had any of the following problems with your work or other regular daily activities **as a result of your physical health?**

(Circle One Number on Each Line)

	Yes	No
13. Cut down the amount of time you spent on work or other activities	1	2
14. **Acomplished less** than you would like	1	2
15. Were limited in the **kind** of work or other activities	1	2
16. Had **difficulty** performing the work or other activities (for example, it took extra effort)	1	2

During the past **4 weeks**, have you had any of the following problems with your work or other regular daily acivities **as a result of any emotional problems** (such as feeling depressed or anxious)?

(Circle One Number on Each Line)

	Yes	No
17. Cut down the **amount of time** you spent on work or other activities	1	2
18. **Accomplished less** than you would like	1	2
19. Didn't do work or other activities as **carefully** as usual	1	2

20. During the **past 4 weeks**, to what extent has your physical health or emotional problems interferred with your normal social activities with family, friends, neighbours or groups?

(Circle One Number)

Not at all	Slightly	Moderately	Quite a bit	Extremely
1	2	3	4	5

21. During the **past 4 weeks**, how much bodily pain have you had?

(Circle One Number)

None	Very mild	Mild	Moderate	Severe	Very severe
1	2	3	4	5	6

22. During the **past 4 weeks**, how much did pain interfere with your normal work (including work both outside the home, and housework?)

(Circle One Number)

Not at all	A little bit	Moderately	Quite a bit	Extremely
1	2	3	4	5

These questions are about how you feel and how things have been with you **during the past 4 weeks**. For each question, please give the one answer that comes closets to the way you have been feeling.

How much of the time during the **past 4 weeks** . . .

(Circle One Number on Each Line)

	All of the time	Most of the time	A good bit of the time	Some of the time	A little of the time	None of the time
23. Did you feel full of life?	1	2	3	4	5	6
24. Have you been nervous?	1	2	3	4	5	6
25. Have you felt so down in the dumps that nothing could cheer you up?	1	2	3	4	5	6
26. Have you felt calm and peaceful?	1	2	3	4	5	6
27. Did you have a lot of energy?	1	2	3	4	5	6
28. Have you felt downhearted and low?	1	2	3	4	5	6
29. Did you feel worn out?	1	2	3	4	5	6
30. Have you been a happy person?	1	2	3	4	5	6

31. Did you feel tired?	1	2	3	4	5	6

32. During the **past 4 weeks**, how much of the time has your **physical health or emotional problems** interfered with your social activities (like visiting with friends, relatives, etc.)?

(Circle One Number)

All of the time	Most of the time	Some of the time	A little bit of the time	None of the time
1	2	3	4	5

How TRUE or FALSE is each of the following statements for you.

(Circle One Number on Each Line)

	Definitely True	Mostly True	Don't Know	Mostly False	Definitely False
33. I seem to get ill more easily than other people	1	2	3	4	5
34. I am as healthy as anybody I know	1	2	3	4	5
35. I expect my health to get worse	1	2	3	4	5
36. My health is excellent	1	2	3	4	5

Loneliness

Please tell me how much of the time during the last week you felt lonely.

None or almost none of the time	Some of the time	Most of the time	All or almost all of the time
1☐	2☐	3☐	4☐

Muscle-strengthening exercise questionnaire (MSEQ)

MSEQ psychometric properties

The Muscle Strengthening Exercise Questionnaire comprised of an explanation of the Chief Medical Officer's guidance on muscle-strengthening exercise and the following two questions …

The UK Department of Health recommends that older adults should do muscle-strengthening exercises on at least two days a week to improve their quality of life and reduce health risks. This means doing exercises (like squats, moving heavy loads like groceries, or repeated lifting of weights) to strengthen your arms, legs and other muscles in your body.

Please circle only one number that you feel best applies to you for each item.

	Strongly disagree	Dis-Agree	Neither agree or disagree	Agree	Strongly agree
I normally do less muscle-strengthening exercise than recommended above.	0	1	2	3	4
I sometimes do the recommended amount of muscle-strengthening exercise.	0	1	2	3	4

The score for item 1 was reversed and then the score for both items was summed to generate the MSEQ Adherence score (total score ranging from 0 to 8). A higher score therefore represented higher adherence. At baseline, the mean MSEQ score was 3.03 (SD 2.07, N=726). Scores ranged from 0 to 8 with an approximately normal distribution and the internal reliability was moderate (Cronbach's Alpha = 0.48, N=0.49).

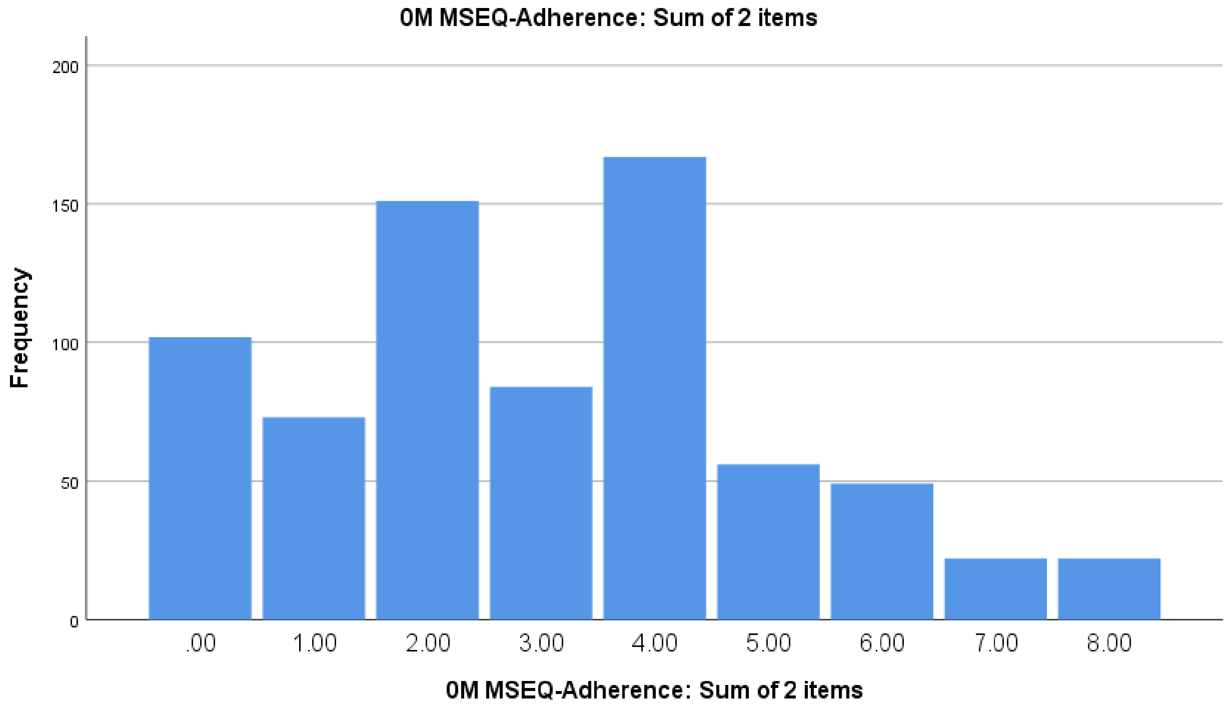

Fig. S.*** Distribution for MSEQ Adherence score at baseline.

Changes in MSEQ adherence score from 0-24 months completely mediated the intervention effect on SPPB at 24 months (Fig. S1), reducing the direct effect to non-significance (p=0.053). The standardised coefficient for the indirect (mediation) path overall was 0.053 (95%CI: 0.020 to 0.094, N=569). Full mediation analyses will be reported in the report to the funder (in the NIHR Public Health Research journal).

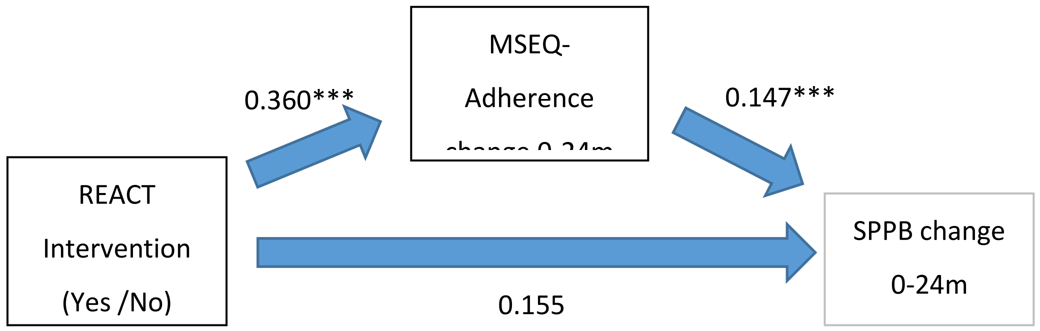

*Figure S1. Mediation of the intervention effect on SPPB at 24 months by change in muscle-strengthening exercise from 0 to 24 months. Age, gender and study site are entered in the model. Figures are standardised coefficients * p<0.05, ** p<0.01, ***p<0.001*

The MSEQ was also demonstrated to be sensitive to change in response to the REACT intervention (which primarily comprised of muscle-strengthening exercises), as demonstrated in the main trial findings (Tables 2, A2, A3).

References

Ageing Well Profile: Stathi, A. & Fox, K. R. (2004). The dimensions of a well-being scale designed for older adults: The ageing-well profile. Journal of Aging and Physical Activity, 12, 300.

The Sleep Condition Indicator: Espie CA, Kyle SD, Hames P, Gardani M, Fleming L, Cape J. The Sleep Condition Indicator: a clinical screening tool to evaluate insomnia disorder. BMJ Open. 2014;4(3).

Pain (Western Ontario and McMaster Universities Osteoarthritis Index (WOMAC): Bellamy N, Buchanan WW, Goldsmith CH, Campbell J, Stitt LW. Validation study of WOMAC: a health status instrument for measuring clinically important patient relevant outcomes to antirheumatic drug therapy in patients with osteoarthritis of the hip or knee. J Rheumatol. 1988;15(12):1833-40.

Euroquol-5 Dimensions (EQ-5D): Herdman M, Gudex C, Lloyd A, Janssen M, Kind P, Parkin D, et al. Development and preliminary testing of the new five-level version of EQ-5D (EQ-5D-5L). Quality of Life Research. 2011;20(10):1727-36.

Short Form-36: Brazier J, Roberts J, Deverill M. The estimation of a preference-based measure of health from the SF-36. Journal of Health Economics. 2002;21(2):271-92.

Loneliness: Yang, K., & Victor, C. (2011). Age and loneliness in 25 European nations. Ageing and society, 31(08), 1368-1388.
